# Comorbidity and temporal associations between mental disorders among college students in the world mental health international college student initiative

**DOI:** 10.1016/j.psychres.2025.116605

**Published:** 2025-06-20

**Authors:** Annelieke M. Roest, Ymkje Anna de Vries, Julia R. Pozuelo, Maria V. Petukhova, Sue Lee, Nancy A Sampson, Yesica Albor, Ahmad N. Alhadi, Jordi Alonso, Nouf Al-Saud, Yasmin Altwaijri, Claes Andersson, Lukoye Atwoli, Randy P. Auerbach, Caroline Ayuya Muaka, Patricia M. Báez-Mansur, Laura Ballester, Jason Bantjes, Harald Baumeister, Marcus Bendtsen, Corina Benjet, Anne H. Berman, Ronny Bruffaerts, Paula Carrasco, Silver C.N. Chan, Irina Cohut, María Anabell Covarrubias Díaz Couder, Marcelo A. Crockett, Pim Cuijpers, Oana A. David, Dong Dong, David D. Ebert, Jorge Gaete, Carlos García Forero, Margalida Gili, Raúl Gutiérrez-García, Josep Maria Haro, Penelope Hasking, Xanthe Hunt, Mathilde M. Husky, Florence Jaguga, Álvaro I. Langer, Irene Léniz, Yan Liu, Scarlett Mac-Ginty, Vania Martínez, Margaret McLafferty, Andrea Miranda, Iris Ruby Monroy-Velasco, Elaine K. Murray, Catherine M. Musyoka, Catalin Nedelcea, Daniel Núñez, Siobhan M. O’Neill, José A. Piqueras, Codruta A. Popescu, Ana Paula Prescivalli, Charlene Rapsey, Kealagh Robinson, Tiscar Rodriguez-Jimenez, Wylene Saal, Oi-ling Siu, Dan J. Stein, Sascha Y. Struijs, Cristina T. Tomoiaga, Karla Patricia Valdés-García, Eunice Vargas-Contreras, Daniel V. Vigo, Angel Y. Wang, Samuel Y.S. Wong, Ronald C. Kessler, World Mental Health International College Student collaborators

**Affiliations:** a Department of Developmental Psychology, Faculty of Behavioral and Social Sciences, University of Groningen, Groningen, the Netherlands; b Department of Child and Adolescent Psychiatry, University Medical Center Groningen, Groningen, the Netherlands; c Department of Global Health and Social Medicine, Harvard Medical School, Boston, MA, USA; d Department of Psychiatry, University of Oxford, Oxford, UK; e Department of Health Care Policy, Harvard Medical School, Boston, MA, USA; f Center for Global Mental Health Research, Instituto Nacional de Psiquiatría Ramón de la Fuente Muñiz, Mexico City, Mexico; g Department of Psychiatry, College of Medicine, King Saud University, Riyadh, Saudi Arabia; h Hospital del Mar Research Institute (IMIM), Barcelona, Spain; i Department of Medicine and Life Sciences, Pompeu Fabra University (UPF), Barcelona, Spain; j Centro de Investigación Biomédica en Red de Epidemiología y Salud Pública (CIBERESP), Instituto de Salud Carlos III, Madrid, Spain; k Biostatistics, Epidemiology and Scientific Computing Department, King Faisal Specialist Hospital and Research Centre, Riyadh, Saudi Arabia; l Department of Criminology, Malmö University, Malmö, Sweden; m Brain and Mind Institute &Medical College of East Africa, the Aga Khan University, Nairobi, Kenya; n Department of Psychiatry, Columbia University, New York, NY, USA; o Department of Psychology & Counselling, Daystar University, Nairobi, Kenya; p Coordinación de Desarrollo Académico y Servicios Educativos, Universidad la Salle Ciudad Victoria, Ciudad Victoria, Mexico; q Mental Health, Alcohol, Substance Use and Tobacco Research Unit, South African Medical Research Council, Cape Town, South Africa; r Department of Psychiatry and Mental Health, University of Cape Town, South Africa; s Department of Clinical Psychology and Psychotherapy, Ulm University, Ulm, Germany; t Department of Health, Medicine and Caring Sciences, Linköping University, Linkoping, Sweden; u Department of Psychology, Uppsala University, Uppsala, Sweden; v Center for Public Health Psychiatry, Katholieke Universiteit Leuven (KUL), Leuven, Belgium; w Campus Gasthuisberg, Universitair Psychiatrisch Centrum KU Leuven (UPC-KUL), Leuven, Belgium; x Unit of Medicine, Faculty of Health Sciences, and FISABIO, Universitat Jaume I (UJI), Castelló de la Plana, Spain; y The Hong Kong University of Science and Technology, Hong Kong SAR; z Career Counseling and Guidance Center, Technical University of Cluj-Napoca, Romania; aa Coordinación de Investigación, Universidad la Salle Noroeste, Ciudad Obregón, Mexico; ab Millennium Nucleus to Improve the Mental Health of Adolescents and Youths (Imhay), Santiago, Chile; ac Faculty of Behavioural and Movement Science, Department of Clinical, Neuro- and Developmental Psychology, Vrije Universiteit Amsterdam, The Netherlands; ad DATA Lab, International Institute for Advanced Studies in Psychotherapy and Applied Mental Health, Babeș-Bolyai University, Cluj-Napoca, Romania; ae Department of Clinical Psychology and Psychotherapy, Babeş-Bolyai University, Cluj-Napoca, Romania; af JC School of Public Health and Primary Care, Faculty of Medicine, The Chinese University of Hong Kong, Hong Kong SAR; ag School of Medicine and Health, Department for Sport and Health Sciences, Technical University Munich, Germany; ah Centro de Investigación en Salud Mental Estudiantil (ISME), Facultad de Ciencias Sociales, Universidad de los Andes, Santiago, Chile; ai Departamento de Medicina, Universitat Internacional de Catalunya (UIC), Barcelona, Spain; aj Department of Psychology, University of the Balearic Islands (UIB), Palma Mallorca, Spain; ak Universidad De La Salle Bajío, Campus Salamanca, Mexico; al Parc Sanitari Sant Joan de Deu, Institut de Recerca Sant Joan de Deu (IRSJD), Sant Boi de Llobregat, Barcelona, Spain; am School of Population Health, Faculty of Health Sciences, Curtin University, Perth, Australia; an Africa Health Research Institute (AHRI), Durban, South Africa; ao Active Team, Bordeaux Population Health Research Center, INSERM U1219, University of Bordeaux, France; ap Department of Alcohol and Drug Abuse Rehabilitation Services, Moi Teaching and Referral Hospital, Eldoret, Kenya; aq Facultad de Psicología y Humanidades, Universidad San Sebastián, Valdivia, Chile; ar Dirección de Salud Mental, Universidad de O’Higgins, Rancagua, Chile; as School of Public Health, Jining Medical University, Jining 272067, Shandong Province, PR China; at Department of Health Service & Population Research, Institute of Psychiatry, Psychology and Neuroscience, King’s College London, London, UK; au ChileCentro de Medicina Reproductiva y Desarrollo Integral del Adolescente (Cemera), Facultad de Medicina, Universidad de Chile, Santiago, Chile; av Personalised Medicine Centre, School of Medicine, Ulster University, Derry, Londonderry, UK; aw Atlantic Technological University, Donegal, Ireland; ax Facultad de Psicología, Universidad Autónoma de Coahuila, Saltillo, Mexico; ay Department of Psychiatry, School of Medicine, University of Nairobi, Nairobi, Kenya; az Department of Psychology and Cognitive Sciences, University of Bucharest, Romania; ba Facultad de Psicología, Universidad de Talca, Talca, Chile; bb School of Psychology, Ulster University, Coleraine, UK; bc Department of Health Psychology, Universidad Miguel Hernandez de Elche (UMH), Alacant, Spain; bd Department of Human Sciences, ‘Iuliu Hatieganu’ University of Medicine and Pharmacy, Cluj-Napoca, Romania; be Department of Psychiatry, Faculty of Medicine, University of British Columbia, Vancouver, BC, Canada; bf Department of Psychological Medicine, University of Otago, Dunedin, New Zealand; bg School of Psychology, Massey University, Wellington, New Zealand; bh Department of Psychology and Sociology, Universidad de Zaragoza (UNIZAR), Zaragoza, Spain; bi Department of Social Sciences, Sol Plaatje University, Kimberley, South Africa; bj Department of Psychology, Lingnan University, Hong Kong, Hong Kong SAR; bk SA MRC Unit on Risk & Resilience in Mental Disorders, University of Cape Town &Neuroscience Institute, Cape Town, South Africa; bl Facultad de Ciencias Administrativas y Sociales, Universidad Autónoma de Baja California, Mexico; bm School of Population and Public Health, Faculty of Medicine, University of British Columbia, Vancouver, BC, Canada

**Keywords:** Internalizing, Externalizing, Comorbidity, College students, Substance use disorders

## Abstract

**Background:**

Mental disorders are highly prevalent among students worldwide. This study aims to examine comorbidity and temporal associations between mental disorders among students.

**Methods:**

The study included 72,288 students from 18 countries as part of the World Mental Health International College Student (WMH-ICS) Initiative, with cross-sectional data collected between 2017 and 2023. Screening for common DSM-5 disorders was conducted using validated screening measures. Latent variables were examined using exploratory principal axis factor analysis on a correlation matrix among the lifetime mental disorders. Based on age-of-onset information, multivariable poisson regression models were used to examine associations of prior disorders with the first onset of other disorders.

**Results:**

27.0 % of students screened positive for only one lifetime disorder, 17.1 % for two, 10.9 % for three, and 10.6 % for 4+ disorders. In the factor analysis, three latent variables were found, comprising: internalizing disorders (generalized anxiety disorder, major depressive episode, post-traumatic stress disorder, and panic disorder), substance use disorders (drug use disorder and alcohol use disorder), and externalizing disorders (attention deficit/hyperactivity disorder and mania/hypomania). Prior internalizing and externalizing disorders were associated with the subsequent first onset of all other disorders with risk ratios ranging from 1.5–7.5. Substance use disorders were less consistently associated with the subsequent first onset of other disorders, but alcohol use disorder was associated with the first onset of drug use disorder and vice versa.

**Conclusions:**

Mental disorder comorbidity is common among students, and students with disorders across the internalizing and externalizing spectrum have an increased risk of future mental disorder comorbidities.

Mental health problems are widespread in the general populations across the world (GBD 2019 Mental Disorders Collaborators, 2022). Mental disorders often have their onset relatively early in life, typically during adolescence or early adulthood (McGorry and van Os, 2013), and tend to be recurrent or chronic. As a result, they frequently have long-term negative consequences (Prince et al., 2007). The issue of mental health problems among young people has become more pressing in recent years, as an increasing number of young people report experiencing high levels of anxiety, depression, and suicidal thoughts (Klaufus et al., 2022). They are also more likely to seek treatment and to report having received a mental health diagnosis, including attention deficit/hyperactivity disorder (ADHD) (Oswalt et al., 2020).

The transition from adolescence to adulthood is a specific period in which mental health problems may emerge, worsen, or recur. Starting college may be a stressful circumstance in a critical period for identity development, development of romantic attachments, becoming independent from parents, and developing social and professional skills (Sussman and Arnett, 2014). Also, student’s health behaviors that could influence their mental health, such as diet, physical activity, and alcohol use may change considerably during this period in life (Almoraie et al., 2025; Bewick et al., 2008; José et al., 2025). Previous studies have examined mental health problems, like depressive and anxiety symptoms, in college students. Yet, in these studies, mental health issues were not based on DSM-5 classifications, nor did they address time-ordered associations in mental disorder comorbidities (Li et al., 2022; Liu et al., 2019).

The World Mental Health International College Student (WMH-ICS) initiative (Auerbach et al., 2016; Cuijpers et al., 2019) involves web-based mental health surveys administered in first-year students in colleges and universities worldwide. Previous publications based on early WMH-ICS surveys reported data on the prevalence and comorbidities of mental disorders for students from eight countries utilizing DSM-IV criteria (Auerbach et al., 2019, 2018). In recent years, the number of participating countries, universities, and students has increased substantially. In addition, the screening for disorders has been expanded to include more diagnoses, all based on DSM-5 criteria. Mason et al. (2025) reported data on the lifetime and 12-month prevalence of these mental disorders in 72,288 respondents. However, no data was presented on comorbidity. The current study on mental disorder comorbidity is one of a series of papers analyzing this sample (Mason et al., 2025), with others investigating non-suicidal self-injury (Hasking et al., 2025); suicidal thoughts and behaviors (Mortier et al., 2025) and associations between childhood adversities and mental disorders (Husky et al., 2025).

Mental disorder comorbidity, defined as experiencing multiple mental disorders simultaneously or sequentially, is very common (Auerbach et al., 2019) and tends to be associated with a severe and persistent course of illness (Roest et al., 2021). For example, mental disorder comorbidity predicts college dropout in college students (Auerbach et al., 2016) and is a predictor of suicidality in young people (Gili et al., 2019). Previous studies also demonstrate that early life mental disorders (such as anxiety disorders and ADHD) are strong predictors of the future onset, persistence, and severity of mental disorders and of the subsequent onset of physical disorders (de Vries et al., 2022, 2019; Meinzer et al., 2013; Roest et al., 2017; Uher et al., 2024). A comprehensive analysis of pair-wise associations using the World Mental Health adult surveys, using information on age-of-onset of disorders, revealed that each mental disorder predicted the subsequent onset of all other mental disorders (McGrath et al., 2020), demonstrating the ubiquity of comorbidity. However, it is unclear whether the patterns of mental disorder comorbidity development are the same in early versus later adulthood (Uher et al., 2024).

The first aim of the current study is to examine the prevalence of mental disorder comorbidity among college students using the expanded WMH-ICS dataset. The second aim is to study the temporal associations between mental disorders using information on age of onset of the disorders.

## 1. Methods

### 1.1. Participants & procedures

Online surveys were carried out in a convenience sample of 77 universities across 18 countries (Australia, Belgium, Canada, Chile, China, France, Germany, Kenya, Mexico, Netherlands, New Zealand, Northern Ireland, Republic of Ireland, Romania, Saudi Arabia, South Africa, Spain, and Sweden). Although the recruitment method varied by institution (Supplementary Table 1), attempts were generally made to recruit 100 % of first-year students via emails provided by participating universities requesting participation in a confidential online survey of student mental health. Participants were provided with a study description, an informed consent script, and a university phone number for questions. Incentives, which differed across countries (e.g., raffles for store credit coupons, movie passes, cash), were offered in 11 of the 18 countries to encourage survey completion (Supplementary Table 1). Informed consent was required before administering the survey. Reminder emails were used to increase response rates. Within-country sample sizes ranged from *n* = 333 in Kenya to *n* = 11,607 in The Netherlands. Ethics approval details are posted at https://www.hcp.med.harvard.edu/wmh/ftpdir/IRB_EthicsApproval_WMH-ICS_DSM-5.pdf

### 1.2. Measures

The self-report questionnaire (https://www.hcp.med.harvard.edu/wmh/ftpdir/WMH-ICS_Baseline_survey_V3.2_FINAL_20220228.pdf) was developed in English and translated into local languages using a translation, back-translation, and harmonization protocol to maximize cross-national equivalence building on the standard World Health Organization (WHO) protocol (Harkness et al., 2008).

#### 1.2.1. Mental disorders

Lifetime prevalence of DSM-5 generalized anxiety disorder (GAD), major depressive episode (MDE), and panic disorder (PD) was assessed with the Composite International Diagnostic Interview Screening Scales, Version 3.2 (CIDI-SC; Kessler et al., 2013a). Diagnoses based on CIDI-SC have been shown to have good concordance with diagnoses based on blinded clinical reappraisal interviews (Kessler et al., 2013a, 2013b). Lifetime assessments of mania/hypomania (M/HM) and drug use disorder (DUD) were based on the Composite International Diagnostic Interview for DSM-5 (CIDI-5) modified for self-report administration. Although only one clinical reappraisal study has assessed CIDI-5 so far, concordance of diagnoses with diagnoses based on blinded clinical reappraisal interviews was consistently good (AU-ROC=0.67–0.75) (Khaled et al., 2024). See Supplementary Table 2 for operational definitions of CIDI-SC and CIDI DSM-5 diagnoses.

The other three disorders, post-traumatic stress disorder (PTSD), ADHD, and alcohol use disorder (AUD) were assessed with brief specialized dimensional screening scales, namely the 4-Item Short-Form of the PTSD Checklist for DSM-5 (PCL-5; Weathers et al., 2013); the Adult Self-Report Scale-V1.1(ASRS-V1.1) Screener for ADHD (Kessler et al., 2005); and the Alcohol Use Disorders Identification Test (AUDIT) (Babor et al., 1992).

The PCL-5 is a widely used and validated PTSD screening scale (Georgescu et al., 2024; Hansen et al., 2023; Kramer et al., 2023). Diagnoses obtained by using a cutpoint of 5+ on the 4-Item Short-Form PCL-5 (each item scored in the range 0–4 for a total score of 0–16) have good concordance with DSM-5 diagnoses in the full PCL-5 (AU-ROC=0.98) (Zuromski et al., 2019).

The ASRS-V1.1 Screener is a widely used and validated 6-item screening scale of adult ADHD (each item scored in the range 0–4 for a total score of 0–24) (Ziobrowski et al., 2023) that assesses symptoms over a 6-month recall period. Diagnoses obtained by using a cutpoint of 14+ have been shown to have good concordance with blinded clinical diagnoses in multiple clinical reappraisal studies (Kessler et al., 2020; Kessler et al., 2013b).

Lastly, the AUDIT, a widely used and validated 10-question screening scale for AUD (each item scored in the range 0–4 for as total score of 0–40), assesses symptoms over a 12-month recall period. We used the standard AUDIT scoring rules for possible dependence (either a score of 16 or more on the 0–40 total AUDIT or a score of 8–15 on the total AUDIT in conjuntion with a score of 4+ on the AUDIT dependence subscale), which have had high concordance with blinded clinical diagnoses of AUD in prior research (AU-ROC=0.91) (Toner et al., 2019). However, as more recent studies suggest that a lower threshold might be preferable for university students, we also included AUDIT scores for likely abuse (8+ on the total AUDIT; Villarosa-Hurlocker et al., 2020).

For the six mental disorders where lifetime prevalence was assessed (GAD, MDE, PD, PTSD, M/HM, DUD), respondents were asked lifetime diagnostic stem questions and then, if affirmative, were asked to focus on the time in their life when the symptoms were most severe. The symptom questions were asked about that worst time, which could differ within respondents across mental disorders. Respondents screening positive for lifetime prevalence were then asked about age-of-onset (AOO) and a single question (i.e., rather than repeating all symptom questions) about 12-month prevalence. ADHD and AUD, in comparison, were assessed only for the past 6 months or 12 months, respectively but respondents were also asked about AOO of ADHD and AUD.

#### 1.2.2. Socio-demographics

The socio-demographics considered here were respondent age (18–36+ years old), sex at birth (male, female), gender identity (male, female, another gender), sexual orientation (heterosexual/straight, gay/lesbian, other), and parent education (highest education of either parent dichotomized into college degree versus less than college degree). Dichotomous variables were created for sexual orientation (heterosexual vs non-heterosexual) and gender modality (cisgender vs transgender, based on alignment between gender identity and sex at birth). As neither gender identity nor sexual orientation was assessed in Saudi Arabia, gender identity was set equal to sex at birth and sexual orientation was set equal to heterosexual in that survey.

### 1.3. Data analysis

A calibration weight was used to adjust for differential within-university response rates by student age and sex at birth. Multiple imputation (MI) by chained equations (Van Buren, 2012) was then used to adjust for within-survey item non-response (0–3 % across items), missing data due to minor skip logic errors in a few surveys, and random internal subsampling of survey sections. The latter was a variation on the split questionnaire design (Raghunathan and Grizzle, 1995) to shorten assessments while still obtaining information about all outcomes from all respondents. We did this in universities where concerns were raised about survey length by administering diagnostic stem questions for four diagnoses with long question series -PD, M/HM, PTSD, and AUD- to all respondents and then administering full diagnostic sections only to a probability subsample of 40 % of the respondents who endorsed the stems. Students who screened negative were coded no. Students who screened positive and had the full assessment were coded either yes or no depending on subsequent question responses. Students who screened positive but were randomized not to have the full assessment (between 13.1 % for M/HM and 21.5 % for AUD across disorders) were assigned predicted probabilities based on MI methods. Cross-validated analyses demonstrate that prevalence estimates were not biased by using MI in this way (Mason et al., 2025).

Simple mean calculations across all 30 imputations, with standard errors [SE]s adjusted for MI data, were used to estimate lifetime prevalence, 12-month prevalence, and 12-month persistence. Persistence analyses focused on respondents whose AOO occurred at least two years prior to age-at-interview, as some proportion of respondents with more recent AOO would necessarily be 12-month cases due to onsets occurring <12 months before interview (e.g., onset near the end of age 18 for a respondent who only recently turned 19 at the time of interview).

We performed exploratory principal axis factor analysis on a correlation matrix among the lifetime mental disorders using promax rotation and person-level data to study latent variables. We selected the best solution using the parallel analysis simulation method (Lim and Jahng, 2019). Multivariable Poisson regression models were used to examine associations of temporally primary disorders with the first onset of each secondary disorder and with 12-month persistence of the secondary disorder. Exponentiated Poisson regression coefficients are reported here as risk ratios (RRs) with 95 % confidence intervals.

The lifetime models were estimated in a person-year discrete-time survival framework where year of life was treated as a continuous control variable, the outcome (i.e., first onset of the outcome disorder) was defined dichotomously, and person-years beyond the year of onset were censored (Singer and Willett, 1993). Persistence models were estimated to predict 12-month prevalence among lifetime cases at the person-level, using AOO and time-since-onset (i.e., number of years between AOO and age-at-survey) as separate control variables and, as noted above, limiting analysis to respondents with AOO 2+ years prior to age-at-interview.

To examine the association between temporally primary disorders and secondary disorders, we included disorders with onset prior to the onset of the outcome disorder (“prior disorders”) and disorders with onset in the same year as the outcome disorder (“same-year disorders”) in the model as separate predictors, as temporal ordering cannot be clearly established for disorders with onset in the same year. In contrast, for the persistence models, we combined these prior and same-year disorders, as both prior and same-year disorders developed temporally primary to current persistence.

Clustering of observations within universities was taken into consideration in estimating SEs. Regression models included control variables for country, year of survey, and whether students were surveyed in the first three months of the academic year, as well as socio-demographic variables previously found to be associated with prevalence (sex at birth, parental education, gender modality [transgender vs. cisgender], and non-heterosexuality), generating pooled within-country/within-year regression coefficients. Disorders assessed only for 6-month (ADHD) or 12-month (AUD) prevalence were excluded from persistence analyses.

For both onset and persistence models, we estimated a sequence of models that included: Model 1) a set of models estimating the association between each individual prior/same-year disorder and the outcome disorder (e.g., prior ADHD predicting onset of MDE); Model 2) a model estimating the association between all prior/same-year disorders and the outcome disorder simultaneously; and Model 3) a model estimating the association between all prior/same-year disorders and the outcome disorder simultaneously, but additionally controlling for number of prior/same-year internalizing, externalizing, and substance use disorders (based on the factor solution). This third model allows for the possibility of sub- or super-additive interactions (i.e. if the effect of an additional disorder depends on how many other disorders a person has).

Design-based SEs and F tests taking into consideration clustering, multiply imputed data, and weighting were used to evaluate statistical significance. Design-based SEs were obtained using SAS (V15.2). Stata/MP (V18) (StataCorp, 2023) was used to estimate multivariable Poisson models with robust variance estimates to adjust for design effects (Chen et al., 2018). All significance tests were evaluated using 0.05-level two-sided design-based tests. The multiple testing problem, which leads to increased risk of Type I errors, was addressed by evaluating significance of full predictor sets in each multivariable model and interpreting individually significant coefficients only if the total model was significant.

## 2. Results

### 2.1. Sample characteristics

The response rates ranged between 2.8 % (Kenya) and 65.4 % (Mexico) with a weighted (by achieved sample size) response rate across countries of 20.8 %. Country specific response rates are presented in Supplementary Table 1.

Respondents had a median age of 19 years (IQR:18–22), with 57.9 % (SE:0.2) assigned as female at birth. Most respondents identified as cisgender (98.6 %, SE:0.0) and heterosexual (79.0 %, SE:0.2). Close to half had at least one parent with a college degree (45.7 %, SE:0.2).

27.0 %, (SE:0.2) of participants had one, 17.1 % (SE:0.2) two, 10.9 % (SE:0.1) three, and 10.6 % (SE:0.1) more than three lifetime mental disorders. 26.8 %, (SE:0.2) of all participants had one, 14.7 % (SE:0.2) two, 8.9 % (SE:0.1) three, and 7.1 % (SE:0.1) more than three 12-month mental disorders.

### 2.2. Exploratory factor analysis

Exploratory principal axis factor analysis identified an optimal 3-factor solution that distinguished internalizing disorders (MDE, PD, GAD, PTSD), substance use disorders (AUD, DUD), and externalizing disorders (M/HM, ADHD) (see Table 1).

### 2.3. Temporally primary disorders and the first onset of other mental disorders

In Model 1, nearly all temporally primary disorders were positively associated with the first onset of other mental disorders. No positive associations were found for: Temporally primary ADHD and the same-year onset of AUD and DUD, and temporally primary AUD and DUD and the onset of ADHD. These exceptions may be explained by the DSM-specified early age of onset of ADHD (<12 years old).

**Fig. 1.**
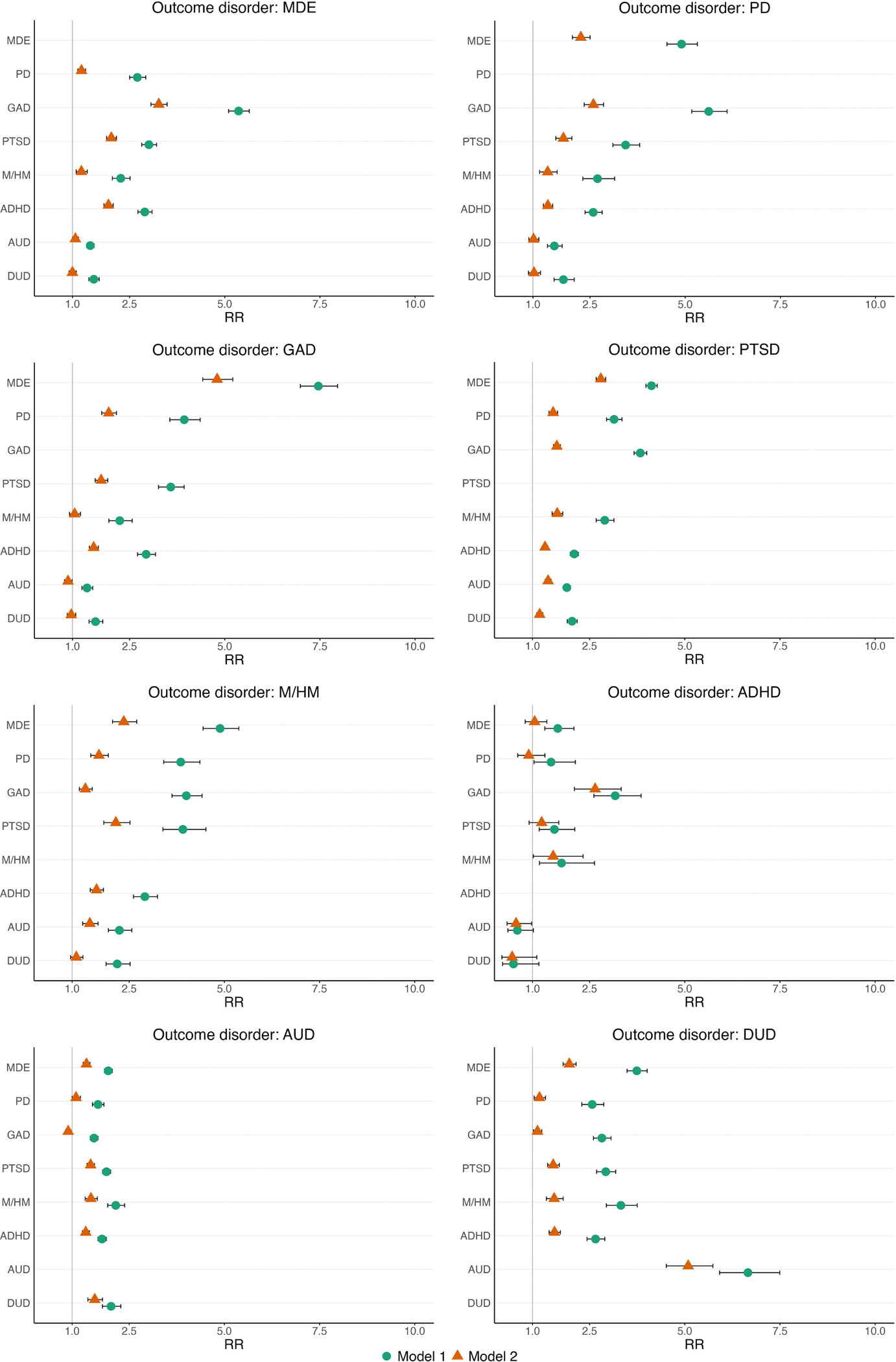
Temporally primary disorders predicting the first onset of other mental disorders Forest plots showing associations (RR and 95 %CI) between prior disorders (age of onset prior to age of onset outcome disorder) and first onset of other mental disorders. Model 1 estimates the association between each individual prior/same-year disorder and the outcome disorder (e.g., prior ADHD and the onset of MDE); Model 2 estimates the association between all prior/same-year disorders and the outcome disorder simultaneously. Both models include controls for country, year of survey, whether students were surveyed in the first three months of the academic year, sex at birth, parental education, gender modality [transgender vs. cisgender], and non-heterosexuality.

Same-year associations were generally stronger than associations for prior disorders with the onset of other mental disorders. Among statistically significant positive associations, RRs for prior disorders and the onset of other disorders ranged from RR=1.4 (95 % CI:1.3–1.5) for prior AUD and the onset of GAD to RR=7.5 (95 % CI:7.0–8.0) for prior MDE and the onset of GAD. For same-year onset associations, RRs ranged from RR=1.5 (95 % CI:1.1–2.0) for ADHD and the onset of PTSD to RR=38.3 (95 %CI:36.2–40.4) for MDE and the onset of GAD (see Table 2 and Fig. 1).

In Model 2, in which all temporally primary disorders were included simultaneously, both prior disorders (as a group) and same-year disorders (as a group) were associated with the onset of all outcome disorders (all p-values <0.001). However, when examining associations between individual disorders, the strength of these associations attenuated compared with model 1, with some associations losing statistical significance (see Fig. 1 and Supplementary Table 3).

In Model 3, we additionally controlled for the number of temporally primary internalizing, externalizing, and substance use disorders. Sub-additive interactions were found since the RRs of the internalizing, externalizing, and substance use scales were generally less than one, indicating that the risk of disorder onset increased at a decreasing rate with the number of temporally primary disorders. However, these sub-additive interactions were not always statistically significant, especially for the substance use scale (see Supplementary Table 4).

### 2.4. Temporally primary disorders and the persistence of other disorders

**Fig. 2.**
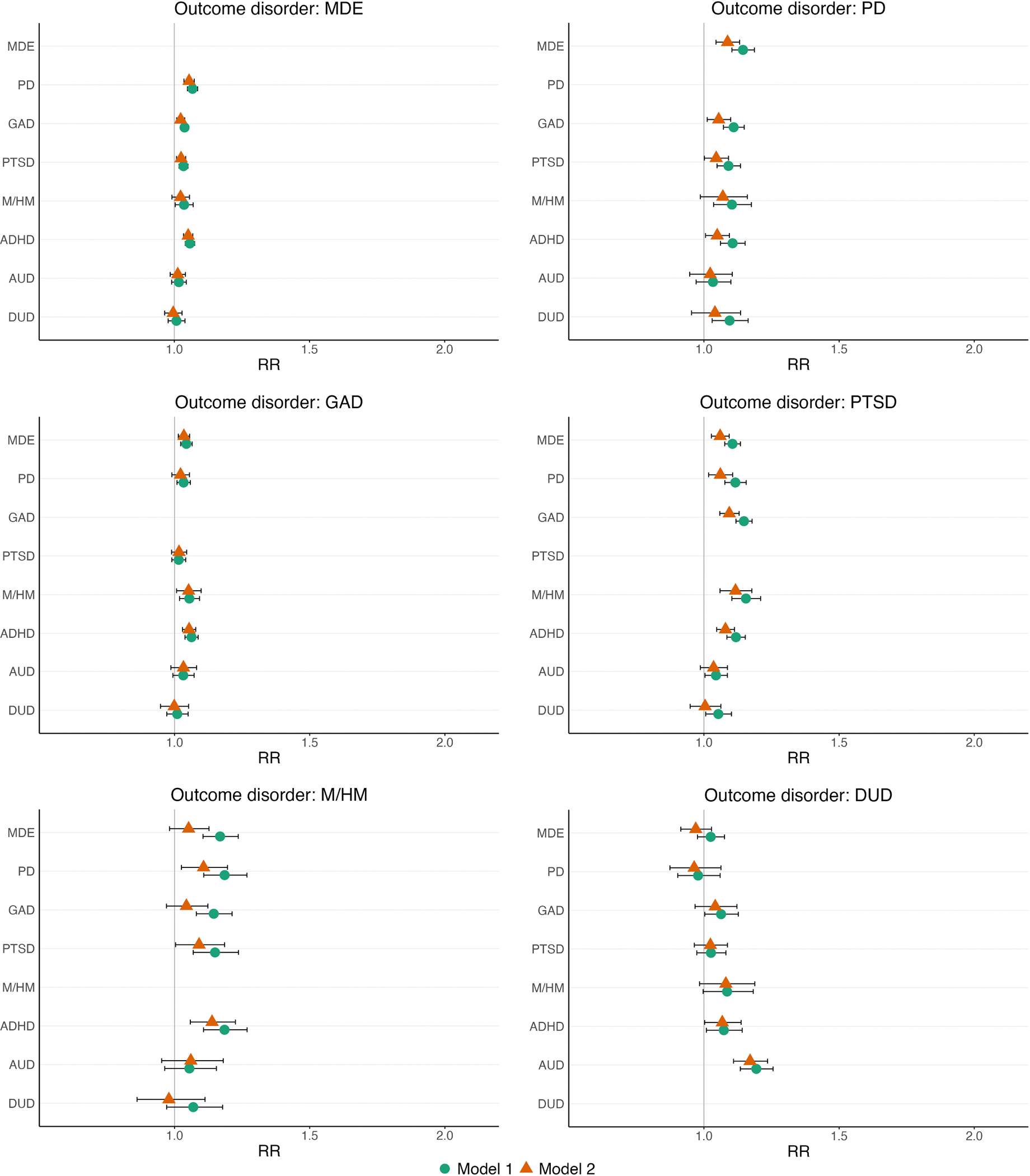
Temporally primary disorders and the persistence of other mental disorders Forest plots showing associations (RR and 95 %CI) between temporally primary disorders and persistence of other mental disorders. Model 1 includes control variables for age of onset and time-since-onset, country, year of survey, whether students were surveyed in the first three months of the academic year, sex at birth, parental education, gender modality [transgender vs. cisgender], and non-heterosexuality). Model 2 additionally adjusts for the presence of other temporally primary disorders.

In model 1, temporally primary GAD and ADHD were significant predictors of persistence for all outcome disorders, with RRs ranging from 1.04 (GAD and the persistence of MDE) to 1.2 (ADHD and the persistence of M/HM) (see Table 3 and Fig. 2). MDE, M/HM, PD, and PTSD also predicted persistence across most outcome disorders, except for DUD and GAD in the case of temporally primary PTSD. In contrast, associations for AUD and DUD with the persistence of other disorders were generally nonsignificant. However, AUD was associated with the persistence of PTSD and DUD, and DUD with the persistence of PTSD and PD.

In model 2, which simultaneously adjusted for all temporally primary disorders, temporally primary disorders (as a group) predicted the persistence of all outcome disorders (all p-values <0.001). However, the strength of the associations for individual disorders attenuated compared to Model 1, with some losing statistical significance (Fig. 2 and Supplementary Table 5).

In model 3, there was no indication of sub-additive interactions, as the RRs for the internalizing, externalizing, and substance use scales with persistence of outcome disorders did not exhibit a consistent pattern of being significantly lower than 1.0. This contrasts with findings for the onset models. However, the associations between temporally primary disorders and persistence of outcome disorders may have been too small to detect sub-additive interactions (see Supplementary Table 6).

## 3. Discussion

This study showed that mental disorder comorbidity is common among college students. While a previous report on the baseline measurement of the WMH-ICS surveys found that 65.2 % of respondents screened positive for lifetime mental disorders, the current study shows that 38.6 % had lifetime comorbidity.

When examining lifetime mental disorders, three latent variables were found: internalizing disorders (MDE, GAD, PD, and PTSD), substance use disorders (DUD and AUD), and externalizing disorders (ADHD and M/HM). Previous studies on the adult WMH surveys also found that bipolar disorder and ADHD loaded on an externalizing factor, which, in contrast to the current study, also encompassed substance abuse (Wardenaar et al., 2018). Temporally primary individual internalizing disorders were associated with the subsequent first onset of other internalizing disorders, as well as M/HM, and substance use disorders. Similarly, temporally primary M/HM and ADHD were associated with the subsequent onset of other externalizing disorders, and substance use disorders, but also internalizing disorders. In contrast, the associations between AUD and DUD with the subsequent first onset of other disorders generally lost statistical significance after adjusting for comorbid disorders, indicating that these associations can be better explained by other comorbid disorders. Yet, AUD ad DUD were associated with each other.. Additionally, we found sub-additive effects, where the risk of disorder onset increased at a decreasing rate with the accumulation of additional disorders. Regarding disorder persistence, temporally primary ADHD was positively associated with the persistence of all other disorders. Furthermore, temporally primary M/HM and internalizing disorders were positively associated with the persistence of other internalizing disorders. Finally, temporally primary AUD was positively associated with the persistence of DUD. However, the statistically significant associations for disorder persistence were considerably weaker (RRs ranging from 1.03 to 1.2) compared to those for disorder onset (RRs ranging from 1.4 to 7.5).

The stronger associations found for disorders with same-year onset (statistically significant associations ranging from 1.5 to 38.3) compared to those with earlier onset (statistically significant associations ranging from 1.4 to 7.5) These findings align with results from a population-based cohort study by Plana-Ripoll and colleagues (2019) that observed higher co-occurrence rates of mental disorders within the first six months after an initial diagnosis, which then diminished over time. There may be several reasons for these findings. The elevated rates could be explained by mental disorders having a broad, interconnected nature, particularly during the early stages of their development (Plana-Ripoll et al., 2019). However, comorbidity in the current study may partly result from recall bias, or may have been overestimated as a result of the use of symptom-based screening measures. Consequently, the strength of (same-year) associations may have been overestimated and therefore should be interpreted with caution. On the other hand, it is also plausible that some individuals will develop several mental disorders within a relatively short amount of time, for example, after encountering stressful or traumatic experiences.

The positive associations between GAD and MDE found in this study are in line with studies examining the structure of psychopathology, which consistently find that GAD and depression both load on a “distress” factor within an internalizing disorder factor (de Jonge et al., 2018; Kotov et al., 2022). Further, our results concur with other studies showing that comorbidity with depression was very high in adults with lifetime or 12-month GAD, namely 52.6 % and 40.9 % respectively (Ruscio et al., 2017). In follow-up data from the National Comorbidity Survey, baseline GAD significantly predicted subsequent MDE onset and persistence, while baseline MDE only significantly predicted subsequent GAD onset (Kessler et al., 2008). In our student sample, both MDE and GAD were associated with the persistence of the other (RR of 1.04 for both associations). Nevertheless, associations were much weaker compared to the strengths of the associations for onset of the other disorder (RR of prior GAD predicting MDE was 5.4 and RR of prior MDE predicting GAD was 7.5). In the general population adult WMH survey data, we found that comorbid anxiety disorders also predict impairment and suicidality in persons with 12-month depression (Roest et al., 2021). Future studies should examine associations between mental disorder comorbidity and suicidality and impairment in college students.

The finding of only few significant associations between the sub stance use disorders with the onset and persistence of other disorders may reflect their typically later onset relative to other disorders, specifically ADHD and anxiety disorders (Solmi et al., 2022). Given that the median age of our sample is 19, alcohol- and drug-related issues are likely to be relatively recent developments. Additionally, alcohol and drug use are common and often normalized within student populations. Therefore, it is possible that the substance use disorder diagnoses in the current study mostly reflect abuse and not necessarily dependence. Substance use problems may decrease for a proportion of the students over the course of college (Bewick et al., 2008) and continue to lessen as they transition into working life.

Clarifying the directionality between the onsets of mental disorders could provide valuable insights for developing prevention and treatment strategies (Cuijpers et al., 2019). In the present study, most temporally, primary disorders were associated with increased risk of onset, and, to a lesser extent, persistence of subsequent disorders. Notably, ADHD was related to both onset and persistence of a wide range of other disorders, likely due in part to its early age of onset. However, early treatment of ADHD may not prevent the onset of secondary disorders. Currently, there is no convincing evidence that interventions for ADHD in childhood are beneficial for ADHD symptomatology and functioning in the long-term (Roest et al., 2023a) In addition, caution is warranted when adopting a “treatment as prevention” approach (Copeland et al., 2022; Roest et al., 2023b), since primary treatments may have unintended negative long-term effects (Copeland et al., 2022; Roest et al., 2023a). Furthermore, whether treatment influences the development of secondary disorders may depend on the type of causal processes underlying the comorbidity (Kessler and Price, 1993). For example, secondary substance use disorder may have developed in students with ADHD as a result of self-medication (Stueber and Cuttler, 2022), or impulsive behavior may have led to AUD (Luderer et al., 2021). ADHD and substance use disorders may also share common genetic determinants (Skoglund et al., 2015). These varying potential causal mechanisms suggest that distinct intervention strategies could be taken, tailored to each pathway (Kessler and Price, 1993). Additional research into intervening on specific underlying mechanisms as well as transdiagnostic approaches (Dalgleish et al., 2020) for treatment and (secondary) prevention are important avenues for further research within college students (Barnett et al., 2021). For example, for students presenting with anxiety and/or depression, transdiagnostic treatments such as the Unified Protocol for Emotional Disorders (Carlucci et al., 2021), could address common underlying mechanisms like rumination, worry, and avoidance (Cuijpers et al., 2023). In addition, it is conceivable that specific groups of students, may benefit from targeted interventions designed to address specific challenges, for example procrastination and stress management in students with ADHD (Cuijpers et al., 2021). Finally, this study highlights that professionals in student mental health services should be vigilant about comorbid mental health disorders among students seeking help. This vigilance could be supported by targeted training for college counseling staff in comorbidity-aware, transdiagnostic frameworks that enable providers to recognize and respond to complex and overlapping symptom presentations (Marchette and Weisz, 2017). In parallel, campus-wide psychoeducation and mental health literacy programs may promote early help-seeking and reduce stigma associated with multi-symptom presentations (Wei et al., 2013). In addition, health behaviors could also serve as prominent targets for improving mental health outcomes in students, for example by educational events promoting active lifestyles and heathy diets (Herbert, 2022; Moscatelli et al., 2023).

This study has several strengths. First, it includes a large sample, including countries for which less data is typically available, providing robust statistical power to examine comorbidity patterns and disorder trajectories. Second, it covers screeners for eight major mental disorders, offering a comprehensive understanding of mental health challenges among college students. Lastly, the inclusion of both lifetime and 12-month prevalence data enables a nuanced analysis of disorder persistence and onset dynamics.

Limitations of this study are the following, first, the WMH-ICS surveys are not representative of the entire student population in each country but rather reflect a convenience sample of colleges and universities. In addition, the sample was biased toward upper-middle and high-income countries. In contrast to students in high-income countries, students in low- and middle-income countries may face more or other stressors, such as familial expectations, with less access to treatment services (Evans-Lacko and Thornicroft, 2019). A second limitation is that disorder and comorbidity prevalence rates may be overestimated as a result of low response rates in the surveys (Mason et al., 2025) and by the use of self-report screening instruments. For example, in assessing potential PTSD, we assessed whether an extremely stressful experience was accompanied by avoidance, negative alterations in cognitions and mood, and intrusion symptoms, but the stressors were not restricted to traumatic events per DSM-5 criterion A (i.e., exposure to actual or threatened death, serious injury, or sexual violence) (APA DSM-5). In addition, it is unclear whether PTSD symptoms caused functional impairments. Similarly, for ADHD we do not know whether ADHD symptoms also reduced the quality of academic or social functioning, nor whether symptoms were present in multiple settings, as required by DSM-5 criteria (APA DSM-5). A third limitation is the cross-sectional study design increasing the risk of recall bias (e.g., participants might misremember the timing of their symptoms or incorrectly attribute them to a different time period). However, given the age of participants in this student sample, the time elapsed between the onset of symptoms and the survey is relatively short compared to previous reports on the adult WMH sample (Kiekens et al., 2023). Fourth, ADHD and AUD could not be used as outcome variables in the persistence models. These disorders were assessed for the past 6 months (ADHD) or 12 months (AUD) since these are the specified recall periods for the well-established scales used to assess these disorders. However, respondents were asked about age of onset of ADHD and AUD, so ADHD and AUD could be used as predictor variables in all models, albeit with the exclusion of unknown remitted lifetime cases. A fifth limitation is that, to reduce interview length, probability subsamples of respondents were not requested to complete certain subsets of questions. Since complete case analysis would decrease the sample size substantially, multiple imputation was used for these cases (Mason et al., 2025). Finally, the amount of socio-demographic information was limited, and therefore some potential confounders, such as socioeconomic status and access to mental health services, were not addressed.

In summary, this study found high comorbidity rates of mental disorders among college students. In addition, results show that students with disorders across the internalizing and externalizing spectrum have an increased risk of future mental disorder comorbidities and could potentially benefit from secondary prevention strategies.

## Supplementary Material

1

2

3

4

5

6

Supplementary materials

Supplementary material associated with this article can be found, in the online version, at doi:10.1016/j.psychres.2025.116605.

## Figures and Tables

**Table 1:** Rotated factor loadings (standardized regression coefficients) for lifetime mental disorders.

	Internalizing disorders	Externalizing disorders	Substance use disorders

Major depressive episode	**0.74**	0.13	0.10
Panic disorder	**0.63**	0.05	−0.00
Generalized anxiety disorder	**0.77**	0.07	−0.00
Post-traumatic stress disorder	**0.62**	0.13	0.13
Mania/hypomania	0.16	**0.61**	0.11
Attention-deficit/hyperactivity disorder	0.06	**0.84**	0.01
Alcohol use disorder	−0.02	0.10	**0.80**
Drug use disorder	0.16	0.03	**0.76**

**Table 2:** Temporally primary disorders and the association with subsequent first onset of other disorders.

Model 1^a^		Outcome disorder							
	
		MDE		PD		GAD		PTSD	
				
		RR	(95 %CI)	RR	(95 %CI)	RR	(95 %CI)	RR	(95 %CI)

**Internalizing disorders**									
MDE	Prior	–	–	4.90	(4.52–5.32)	7.46	(6.99–7.96)	4.12	(3.98–4.27)
	Same-year	–	–	9.35	(8.50–10.29)	38.25	(36.18–40.43)	6.16	(5.83–6.50)
PD	Prior	2.70	(2.50–2.92)	–	–	3.94	(3.56–4.35)	3.14	(2.94–3.35)
	Same	7.32	(6.75–7.93)	–	–	14.51	(13.29–15.84)	4.04	(3.67–4.45)
GAD	Prior	5.36	(5.10–5.64)	5.62	(5.17–6.10)	–	–	3.83	(3.67–4.00)
	Same-year	22.71	(21.85–23.60)	15.96	(14.54–17.53)	–	–	6.45	(6.06–6.86)
PTSD	Prior	3.01	(2.82–3.21)	3.43	(3.10–3.80)	3.58	(3.26–3.93)	–	–
	Same	6.58	(6.18–7.00)	4.81	(4.29–5.38)	8.17	(7.52–8.87)	–	–
**Externalizing disorders**									
M/HM	Prior	2.27	(2.05–2.51)	2.70	(2.31–3.15)	2.24	(1.96–2.57)	2.90	(2.67–3.14)
	Same-year	5.53	(4.87–6.26)	5.29	(4.19–6.66)	5.75	(4.85–6.80)	3.72	(3.25–4.25)
ADHD	Prior	2.90	(2.72–3.09)	2.58	(2.37–2.82)	2.93	(2.71–3.18)	2.09	(1.99–2.20)
	Same-year	3.34	(2.87–3.88)	2.65	(1.90–3.72)	5.93	(5.01–7.02)	1.48	(1.12–1.96)
**Substance use disorders**									
AUD	Prior	1.47	(1.37–1.57)	1.56	(1.38–1.77)	1.38	(1.25–1.53)	1.90	(1.81–2.00)
	Same-year	2.47	(2.24–2.74)	2.18	(1.74–2.73)	2.34	(2.03–2.70)	2.56	(2.35–2.78)
DUD	Prior	1.56	(1.43–1.70)	1.80	(1.55–2.08)	1.61	(1.44–1.80)	2.04	(1.91–2.17)
	Same-year	4.64	(4.21–5.12)	4.19	(3.46–5.06)	4.50	(3.92–5.16)	3.09	(2.74–3.48)
Model 1^a^		Outcome disorder							
		M/HM		ADHD		AUD		DUD	
		RR	(95 % CI)	RR	(95 %CI)	RR	(95 %CI)	RR	(95 %CI)
**Internalizing disorders**									
MDE	Prior	4.89	(4.44–5.38)	1.66	(1.32–2.09)	1.95	(1.85–2.06)	3.74	(3.48–4.01)
	Same-year	6.48	(5.62–7.48)	6.67	(5.66–7.85)	2.65	(2.39–2.93)	5.61	(5.05–6.24)
PD	Prior	3.85	(3.41–4.36)	1.49	(1.04–2.12)	1.69	(1.53–1.84)	2.57	(2.29–2.87)
	Same-year	5.44	(4.36–6.90)	4.29	(3.09–5.95)	2.24	(1.80–2.79)	4.51	(3.72–5.46)
GAD	Prior	4.00	(3.62–4.42)	3.17	(2.61–3.85)	1.57	(1.48–1.68)	2.82	(2.60–3.06)
	Same-year	6.13	(5.14–7.30)	8.05	(6.83–9.49)	2.41	(2.10–2.76)	4.94	(4.29–5.69)
PTSD	Prior	3.91	(3.39–4.52)	1.57	(1.18–2.11)	1.90	(1.80–2.01)	2.92	(2.68–3.18)
	Same-year	4.43	(3.77–5.21)	2.46	(1.84–3.28)	2.70	(2.46–2.95)	3.62	(3.16–4.14)
**Externalizing disorders**									
M/HM	Prior	–	–	1.76	(1.18–2.63)	2.15	(1.94–2.38)	3.32	(2.94–3.75)
	Same-year	–	–	2.88	(1.80–4.62)	3.73	(3.20–4.33)	5.08	(4.18–6.19)
ADHD	Prior	2.91	(2.61–3.24)	–	–	1.79	(1.68–1.90)	2.65	(2.43–2.90)
	Same-year	1.90	(1.18–3.05)	–	–	0.16	(0.06–0.45)	0.22	(0.07–0.68)
**Substance use disorders**									
AUD	Prior	2.24	(1.95–2.57)	0.60	(0.35–1.02)	–	–	6.66	(5.91–7.49)
	Same-year	3.89	(3.30–4.57)	0.35	(0.13–0.99)	–	–	11.03	(9.95–12.23)
DUD	Prior	2.18	(1.89–2.52)	0.50	(0.21–1.17)	2.02	(1.80–2.28)	–	–
	Same-year	4.91	(4.02–6.00)	0.50	(0.16–1.53)	8.59	(7.87–9.38)	–	–

ADHD, attention deficit/hyperactivity disorder; AUD, alcohol use disorder; CI, confidence interval; DUD, drug use disorder; GAD, generalized anxiety disorder; MDE, major depressive episode; M/HM, mania or hypomania; PD, panic disorder; PTSD, post-traumatic stress disorder; RR, risk ratio.

a This table displays the associations between disorders with onsets prior to the onset of the outcome disorder (“prior disorders”) and disorders with onset in the same year as the outcome disorder (“same-year disorders”) included as separate predictors in model 1, including control variables for country, year of survey, whether students were surveyed in the first three months of the academic year, as well as socio-demographic variables previously found to be associated with prevalence (sex at birth, parental education, gender modality [transgender vs. cisgender], and non-heterosexuality.

**Table 3:** Temporally primary disorders and the association with persistence of other disorders.

Model 1^a^	Outcome disorder												
		MDE		PD		GAD		PTSD		M/HM		DUD	
						
Predictor	Timing	RR	(95 %CI)	RR	(95 %CI)	RR	(95 %CI)	RR	(95 %CI)	RR	(95 % CI)	RR	(95 %CI)

**Internalizing disorders**													
MDE	Prior or same	–	–	1.14	(1.10–1.19)	1.04	(1.02–1.07)	1.11	(1.08–1.13)	1.17	(1.11–1.24)	1.02	(0.98–1.08)
PD	Prior or same	1.07	(1.05–1.09)	–	–	1.03	(1.01–1.06)	1.12	(1.08–1.16)	1.19	(1.11–1.27)	0.98	(0.90–1.06)
GAD	Prior or same	1.04	(1.02–1.05)	1.11	(1.07–1.15)	–	–	1.15	(1.12–1.18)	1.15	(1.08–1.21)	1.06	(1.00–1.13)
PTSD	Prior or same	1.03	(1.02–1.05)	1.09	(1.05–1.14)	1.02	(0.99–1.04)	–	–	1.15	(1.07–1.24)	1.03	(0.97–1.08)
**Externalizing disorders**													
M/HM	Prior or same	1.04	(1.00–1.07)	1.10	(1.04–1.18)	1.05	(1.02–1.09)	1.16	(1.10–1.21)	–	–	1.09	(1.00–1.18)
ADHD	Prior or same	1.06	(1.04–1.07)	1.11	(1.06–1.15)	1.12	(1.08–1.15)	1.12	(1.08–1.15)	1.18	(1.11–1.27)	1.07	(1.01–1.14)
**Substance use disorders**													
AUD	Prior or same	1.02	(0.99–1.04)	1.03	(0.97–1.10)	1.03	(0.99–1.07)	1.04	(1.00–1.09)	1.05	(0.96–1.16)	1.19	(1.13–1.26)
DUD	Prior or same	1.01	(0.98–1.04)	1.09	(1.03–1.16)	1.01	(0.97–1.05)	1.05	(1.01–1.10)	1.07	(0.97–1.18)	–	–

ADHD, attention deficit/hyperactivity disorder; AUD, alcohol use disorder; CI, confidence interval; DUD, drug use disorder; GAD, generalized anxiety disorder; MDE, major depressive episode; M/HM, mania or hypomania; PD, panic disorder; PTSD, post-traumatic stress disorder; RR, risk ratio.

a This table shows the associations between temporally primary disorders (combining prior and same-year onsets as for outcome disorder) with the current persistence (12-month prevalence among lifetime cases) of outcome disorders. Analyses were adjusted for age of onset and time-since-onset, country, year of survey, whether students were surveyed in the first three months of the academic year, sex at birth, parental education, gender modality [transgender vs. cisgender], and non-heterosexuality.

## Data Availability

The data analyzed in this study is subject to the following licenses/restrictions: The WMH-ICS data sharing agreement limits access of this data to members of the consortium. Requests to access these datasets should be directed to RCK.
