# Supplementary material for: Comorbidity and temporal associations between mental disorders among college students in the world mental health international college student initiative
Source: Psychiatry Res. Author manuscript; Available in PMC 2026 May 18. (PMC13181139; doi:10.1016/j.psychres.2025.116605)
Supplement: 1 [file NIHMS2168631-supplement-1.docx]

Supplementary Material

***Comorbidity and temporal associations between mental disorders among college students in the World Mental Health International College Student Initiative***

Roest et al.

**Supplementary Table 1: WMH-ICS^a^ Sample characteristics**

| **Country** | **Number of participating universities** | **Total size of the universities** | **Number of first-year students (18 +) eligible** | **Number of first-year students (18 +) participated** | **Response rate** | **Data Collection dates** | **Sampling and recruitment procedures^b^** |
| --- | --- | --- | --- | --- | --- | --- | --- |
| **Australia** | 1 public | ~ 52,000 | 26,445 | 3,139 | 11.9% | 2018–2023 | All onshore first-year students were invited to participate through e-mail. Five reminder emails were sent with personalized links to the survey. Conditional incentives were applied (movie passes). |
| **Belgium** | 2 public | ~ 61,000 | 24,615 | 4,899 | 19.9% | 2018–2022 | All eligible first-year students were invited by email (student email) to participate, with up to 7 reminder emails sent to non-respondents. No incentives were provided. |
| **Canada** | 1 public | ~ 58,000 | 7,082 | 1,199 | 16.9% | 2020–2021 | Email invitations were sent weekly to a stratified random sample (based on gender, year of study, age, international student status, degree type) of 350 new students selected from all enrolled students. Two email reminders and a phone call or personalized email reminder (to a randomly selected subgroup of 70 non-responders) were implemented each week. Each respondent was entered into a gift card prize drawing (CAD $1000 = USD $716.70) as incentive. |
| **Chile** | 3 public  2 private | ~ 175,600 | 20,836 | 7,225 | 34.7% | 2020–2021 | All first-year students were invited to participate via email and social media. Between four and eleven reminder emails were sent with the link to the survey. No incentives were used. |
| **China** (Separate samples in Hong  Kong Special Administrative  Region [SAR] and in mainland  China) | 3 public | ~ 31,000 | 18,522 | 2,674 | 14.4% | 2017-23 | All first-year students were invited to participate by email and campus posters with QR codes (Hong Kong only). After verification by the research team of the email addresses, individual survey links were sent to the students. Up to 5 reminder emails, each 1 week apart, were sent. In Hong Kong, all respondents who completed surveys were given HKD100 (=USD 12.8) shopping coupons as incentives. In China, PRC, 10% of the participants received incentives of either RMB100 or RMB50. |
| **France** | 1 public | ~54,000 | 43,701 | 3,640 | 8.3% | 2017–2021 | All first-year students were invited to participate through e-mail. Three reminder emails were sent with personalized links to the survey. A lottery was used to provide incentives (USB flash drives, gift-cards). |
| **Germany** | 2 public | ~ 50,000 | 15,431 | 2,378 | 15.4% | 2018–2021 | All first-year students were invited to participate through e-mail. Six reminder emails were sent with personalized links to the survey. Conditional incentives were applied (store credit coupons). |
| **Kenya** | 2 public  1 private | ~ 148,400 | 11,787 | 333 | 2.8% | 2019–2020 | All first-year students were invited to partcipate through email. Qualtrics software was used to send the invitations. Reminders were sent via email every 3 days. No incentives were provided. |
| **Mexico** | 3 public  4 private | ~ 98,000 | 13,019 | 8,521 | 65.4% | 2018–2023 | All registered first-year students were invited. Initial contact differed by university: students were invited through promotion on social media and by teachers (1 university), or through the department coordinators and tutors who provided time and space to respond either in computer rooms, in classes or on their own time (6 universities). No incentives were provided. |
| **Netherlands** | 6 public | ~ 200,000 | 140,008 | 11,607 | 8.3% | 2018–2022 | All students enrolled in an undergraduate or Master’s program were invited via e-mail to complete the survey for the first 3 cohorts (2018-2020) and in 2021-22, all first-year students were invited. In 2018-2019, after the initial email, reminders were sent to non-responders at least once, but in most cases several times for 14 months. In 2020-22, one initial e-mail and two reminder e-mails were sent. No incentives were applied. |
| **New Zealand** | 1 public | ~ 24,000 | 8,663 | 2,418 | 27.9% | 2021–2022 | All first-year students were invited to participate through student email. Qualtrics software was used to send the invitations. In 2021, one follow-up email was sent; and all Māori (indigenous people), all Pacifica people, and a random sample of 500 non-Māori and non-Pacifica were offered $50.00 to participate. In 2022, four follow-up emails were sent and all Māori (indigenous people), all Pacifica people, and a random sample of 500 non-Māori and non-Pacifica and a random sample of students not living in university residential accommodation were offered $50.00 to participate. |
| **Northern Ireland ^c^** | 1 public | ~ 25,000 | 5,820 | 1,469 | 25.2% | 2019 | All first-year students due to register at 1 college and students from 3 courses at a second college were invited to participate by email. Following registration, ID numbers and links to the survey were provided. Four reminder emails/text messages were sent with personalized links to the survey. All respondents received a university branded hoodie for participating. |
| **Republic of Ireland ^c^** | 1 public | ~ 4,000 | 859 | 360 | 41.9 % | 2019 |  |
| **Romania** | 4 public | ~ 108,000 | 22,165 | 1,577 | 7.1% | 2021 | All first-year students were invited to participate via email where the survey link was provided. Three recruitment campaigns were conducted, with 4 reminder emails sent during each round of recruitment. All respondents were entered into a lottery to win vouchers for online stores and coaching sessions. |
| **Saudi Arabia** | 1 public | ~ 72,200 | 21,231 | 1,230 | 5.8% | 2022–2023 | In the 2022–2023 academic year, all university students were invited to complete the survey by email and Blackboard, an online learning platform. The survey was circulated following a social media mental health campaign. A total of one initial email and three reminders were sent. The university academic affairs department assisted by distributing the survey through email, university newsletter and social media. Respondents were entered in a random drawing of incentives (bookstore gift-cards, -US$100).  In the 2023–2024 academic year, only first-year students were invited to participate. Students received 1 initial email and 2 follow-ups. University faculty supported the study by giving students some time to fill out the survey during their class. The same incentives were also provided. |
| **South Africa** | 24 public | ~657,400 | 135,135 | 14,475 | 10.6% | 2020 | All under-graduate students were invited to participate through e-mail. Three reminder emails were sent with personalized links to the survey. Conditional incentives were applied (5x R1000 draw). |
| **Spain** | 5 public  2 private | ~ 76,900 | 17,659 | 906 | 5.1% | 2020–2022 | In 5 public universities, all undergraduate students were eligible. Invitation methods included: information in the classrooms, poster campaigns, university website, social media. In three universities and some colleges of the fourth, students were invited via email from university authorities, with one to three reminder emails. No monetary incentives were provided. After completing the survey, all participants could download a personalized report with a summary of their results and a list of available resources.  In 2 private universities, first-year students in the schools of nursing and medicine were invited to participate via email from university authorities. Additionally, students were informed about the study in the classrooms. A specific time was allocated to facilitate participation. No incentives were provided. |
| **Sweden** | 7 public | ~ 220,000 | 40,094 | 4,238 | 10.6% | 2020–20 23 | All first-year students with email addresses registered by the university records were invited. The initial invitation email and two reminder emails were sent to students. All emails included a personalized link to the survey. No incentives were provided. |
| **Total** | **77**  **68 public**  **9 private** | **~ 2,115,500** | **573,072** | **72,288** | **20.8%^d^** | **2017-–2023** |  |

^a^ WMH-ICS, World Mental Health Surveys International College Student initiative.

^b^ In cases where the survey was distributed to students in all years of undergraduate or higher level courses, only data from first year undergraduate students (aged 18 +) was included in the cross-national dataset.

^c^ These surveys were coordinated in Northern Ireland and administered in two2 countries.

^d^ Average response rate weighted by achieved sample size
