# Supplementary material for: Comorbidity and temporal associations between mental disorders among college students in the world mental health international college student initiative
Source: Psychiatry Res. Author manuscript; Available in PMC 2026 May 18. (PMC13181139; doi:10.1016/j.psychres.2025.116605)
Supplement: 2 [file NIHMS2168631-supplement-2.docx]

| **Supplementary Table 2. Operational definitions of disorders^a^** |
| --- |
|  |
| **I. DSM-5 Major depressive disorder** |
| **Criterion A:**  Five (or more) symptoms present during the same 2-week period and represent a change from previous functioning, with at least one symptom being either (1) depressed mood or (2) loss of interest or pleasure.  **WMH-ICS Operationalization:** (Five or more responses in (E1a or E1b), E1c-E1d, E2a-E2f = “all or almost all of the time” or “most of the time”) AND (E1a or E1b or E1c = “all or almost all the time” or “most of the time”) |
|  |
| **Criterion B:**  The symptoms cause clinically significant distress or impairment in social, occupational, or other important areas of functioning.  **WMH-ICS Operationalization:** E2g = “all or almost all the time” or “most of the time” |
|  |
| **Criterion C:**  The episode is not attributable to the physiological effects of a substance or to another medical condition.  **WMH-ICS Operationalization:** Not operationalized |
|  |
| **Criterion D:**  The occurrence of the major depressive episode is not better explained by schizoaffective disorder, schizophrenia, schizophreniform disorder, delusional disorder, or other specified and unspecified schizophrenia spectrum and other psychotic disorders.  **WMH-ICS Operationalization:** Not operationalized |
|  |
| **Criterion E:**  There has never been a manic episode or hypomanic episode.  **WMH-ICS Operationalization:** CIDI-5/DSM-5 Bipolar I/II disorder = no |
|  |
| **II. DSM-5 Generalized anxiety disorder** |
| **Criterion A:**  Excessive anxiety and worry (apprehensive expectation), occurring more days than not for at least 6 months, about a number of events or activities (such as work or school performance).  **WMH-ICS Operationalization:** (E7a = “just about every day” or “more days than not”) AND (E7b = “just about every day” or “more days than not”) AND (E7c or E7d = “just about every day” or “more days than not”) |
|  |
| **Criterion B:**  The individual finds it difficult to control the worry.  **WMH-ICS Operationalization:** E7e = “just about every day” or “more days than not” |
|  |
| **Criterion C:**  The anxiety and worry are associated with three (or more) symptoms (with at least some symptoms having been present for more days than not for the past 6 months).  **WMH-ICS Operationalization:** Three or more responses in E8a-E8f = “just about every day” or “more days than not” |
|  |
| **Criterion D:**  The anxiety, worry, or physical symptoms cause clinically significant distress or impairment in social, occupational, or other important areas of functioning.  **WMH-ICS Operationalization:** E8g = “just about every day” or “more days than not” |
|  |
| **Criterion E:**  The disturbance is not attributable to the physiological effects of a substance or to another medical condition.  **WMH-ICS Operationalization:** Not operationalized |
|  |
| **III. DSM-5 Panic disorder** |
| **Criterion A:**  Recurrent unexpected panic attacks. A panic attack is an abrupt surge of intense fear or intense discomfort that reaches a peak within minutes, and during which time four (or more) symptoms occur.  **WMH-ICS Operationalization:** [(E13 = 3 or more and E15 = “all of your attacks occurred without provocation”) OR (E16 = 3 or more)] AND (Four or more responses selected in E14) |
|  |
| **Criterion B:**  At least one of the attacks has been followed by 1 month (or more) of either: 1) Persistent concern or worry about additional panic attacks or their consequences; 2) A significant maladaptive change in behavior related to the attacks.  **WMH-ICS Operationalization:** E17 or E18 = “yes” |
|  |
| **Criterion C:**  The disturbance is not attributable to the psychological effects of a substance or another medical condition.  **WMH-ICS Operationalization:** Not operationalized |
|  |
| **Criterion D:**  The disturbance is not better explained by the symptoms of another mental disorder.  **WMH-ICS Operationalization:** Not operationalized |
|  |
| **IV. CIDI-5/DSM-5 Bipolar I/II disorder** |
| Criteria have been met for at least one manic episode.  **WMH-ICS Operationalization:** (E24a or E24b = “all or almost all the time” or “most of the time”) AND (One or more responses in E25a-E25e = “all or almost all the time” or “most of the time”) AND (E29 = “7-14 days” or “15 or more days” OR E32 = “yes”) AND [(E24a = missing or “some of the time” or “a little of the time” or “none of the time” AND E24b = “all or almost all the time” or “most of the time” AND four or more responses in E24c, (E25a-E25e), E26a-e = “all or almost all the time” OR “most of the time”) OR (E24a = “all or almost all the time” or “most of the time” AND three or more responses in E24c, (E25a-E25e), E26a-e = “all or almost all the time” OR “most of the time”)] AND (E30 = “extremely” or “a lot” OR E32 = “yes”)  OR  There has never been a manic episode but criteria have been met for at least one major depressive episode and at least one hypomanic episode.  **WMH-ICS Operationalization:** (CIDI-5/DSM-5 Bipolar I disorder = no) AND (DSM-5 Major depressive disorder = yes) AND (E24a or E24b = “all or almost all the time” or “most of the time”) AND (One or more responses in E25a-E25e = “all or almost all the time” or “most of the time”) AND (E29 = “4-6 days” OR “7-14 days” OR “15 or more days”) AND [(E24a = missing or “some of the time” or “a little of the time” or “none of the time” AND E24b = “all or almost all the time” or “most of the time” AND four or more responses in E24c, (E25a-E25e), E26a-e = “all or almost all the time” OR “most of the time”) OR (E24a = “all or almost all the time” or “most of the time” AND three or more responses in E24c, (E25a-E25e), E26a-e = “all or almost all the time” OR “most of the time”)] AND (B9b = “yes” OR E23 = “yes”) AND (E31 = “often” OR “sometimes”) AND (E30 is NOT “extremely” or “a lot” AND E32 is NOT “yes”) |
|  |
| **V. DSM-5 Drug use disorder** |
| **Criterion A:**  A problematic pattern of use of an intoxicating substance leading to clinically significant impairment or distress, as manifested by at least two symptoms, occurring within a 12-month period.  **WMH-ICS Operationalization:** Two or more responses in F12a-e, F13a-f = “every or nearly every day” OR “3-4 days a week” OR “1-2 days a week” OR “1-3 days a month” |
|  |

Abbreviations: DSM-5, Diagnostic and Statistical Manual of Mental Disorders, 5^th^ edition; WMH-ICS, World Mental Health International College Student Initiative.

^a^Item numbers refer to the numbering in the DSM-5 WMH-ICS Version 3 Questionnaire, which is posted at <https://www.hcp.med.harvard.edu/wmh/ftpdir/WMH-ICS_Baseline_survey_V3.2_FINAL_20220228.pdf>
