# Supplementary material for: Comorbidity and temporal associations between mental disorders among college students in the world mental health international college student initiative
Source: Psychiatry Res. Author manuscript; Available in PMC 2026 May 18. (PMC13181139; doi:10.1016/j.psychres.2025.116605)
Supplement: 3 [file NIHMS2168631-supplement-3.docx]

| **Supplementary Table 3. Temporally primary disorders (prior or same age of onset) and subsequent first onset of other mental disorders**  **Model 2** | | | | | | | | | | | | | | | | | |
| --- | --- | --- | --- | --- | --- | --- | --- | --- | --- | --- | --- | --- | --- | --- | --- | --- | --- |
|  |  |  | **MDE** | | |  | **M/HM** | | |  | **Panic** | | |  | **GAD** | | |
| **Predictor** | **Timing** |  | **RR** | **Lower bound** | **Upper bound** |  | **RR** | **Lower bound** | **Upper bound** |  | **RR** | **Lower bound** | **Upper bound** |  | **RR** | **Lower bound** | **Upper bound** |
| MDE | Prior |  | - | - | - |  | 2.4 | 2.1 | 2.7 |  | 2.3 | 2.0 | 2.5 |  | 4.8 | 4.4 | 5.2 |
|  | Same |  | - | - | - |  | 3.5 | 3.0 | 4.1 |  | 3.7 | 3.3 | 4.2 |  | 23.9 | 22.3 | 25.7 |
| M/HM | Prior |  | 1.2 | 1.1 | 1.4 |  | - | - | - |  | 1.4 | 1.2 | 1.6 |  | 1.1 | 0.9 | 1.2 |
|  | Same |  | 2.3 | 2.0 | 2.7 |  | - | - | - |  | 2.3 | 1.8 | 2.9 |  | 1.8 | 1.5 | 2.1 |
| Panic disorder | Prior |  | 1.2 | 1.1 | 1.3 |  | 1.7 | 1.5 | 2.0 |  | - | - | - |  | 2.0 | 1.8 | 2.2 |
|  | Same |  | 2.1 | 1.9 | 2.4 |  | 2.3 | 1.8 | 2.9 |  | - | - | - |  | 4.4 | 4.0 | 4.8 |
| GAD | Prior |  | 3.3 | 3.1 | 3.5 |  | 1.3 | 1.2 | 1.5 |  | 2.6 | 2.3 | 2.9 |  | - | - | - |
|  | Same |  | 12.9 | 12.2 | 13.7 |  | 2.1 | 1.7 | 2.5 |  | 6.5 | 5.7 | 7.3 |  | - | - | - |
| PTSD | Prior |  | 2.0 | 1.9 | 2.2 |  | 2.1 | 1.8 | 2.5 |  | 1.8 | 1.6 | 2.0 |  | 1.8 | 1.6 | 1.9 |
|  | Same |  | 3.7 | 3.5 | 4.0 |  | 2.7 | 2.3 | 3.2 |  | 2.4 | 2.1 | 2.7 |  | 3.0 | 2.7 | 3.2 |
| ADHD | Prior |  | 1.9 | 1.8 | 2.1 |  | 1.6 | 1.5 | 1.8 |  | 1.4 | 1.3 | 1.5 |  | 1.6 | 1.4 | 1.7 |
|  | Same |  | 2.3 | 1.9 | 2.6 |  | 1.7 | 1.1 | 2.8 |  | 1.9 | 1.4 | 2.7 |  | 3.8 | 3.1 | 4.6 |
| AUD | Prior |  | 1.1 | 1.0 | 1.2 |  | 1.5 | 1.3 | 1.7 |  | 1.0 | 0.9 | 1.2 |  | 0.9 | 0.8 | 1.0 |
|  | Same |  | 1.7 | 1.5 | 1.9 |  | 2.6 | 2.2 | 3.0 |  | 1.4 | 1.1 | 1.7 |  | 1.2 | 1.1 | 1.4 |
| DUD | Prior |  | 1.0 | 0.9 | 1.1 |  | 1.1 | 1.0 | 1.3 |  | 1.0 | 0.9 | 1.2 |  | 1.0 | 0.9 | 1.1 |
|  | Same |  | 2.3 | 2.0 | 2.5 |  | 2.1 | 1.7 | 2.6 |  | 1.9 | 1.6 | 2.3 |  | 1.6 | 1.4 | 1.8 |
|  |  |  |  |  |  |  |  |  |  |  |  |  |  |  |  |  |  |
| **Significance tests of predictors** | |  | **F-value** | **p-value** | **Numerator DF** |  | **F-value** | **p-value** | **Numerator DF** |  | **F-value** | **p-value** | **Numerator DF** |  | **F-value** | **p-value** | **Numerator DF** |
| All dx | Prior |  | 602.9 | 0 | 7 |  | 226.1 | 0 | 7 |  | 318.4 | 0 | 7 |  | 476.1 | 0 | 7 |
| All dx | Same |  | 2601.3 | 0 | 7 |  | 183.2 | 0 | 7 |  | 614.0 | 0 | 7 |  | 2543.2 | 0 | 7 |
|  |  |  |  |  |  |  |  |  |  |  |  |  |  |  |  |  |  |

ADHD, attention deficit/hyperactivity disorder; AUD, alcohol use disorder; DUD, drug use disorder; GAD, generalized anxiety disorder; MDE, major depressive episode; M/HM, mania or hypomania; PTSD, post-traumatic stress disorder; RR, risk ratio

| **Supplementary Table 3 (continued). Temporally primary disorders (prior or same age of onset) predicting subsequent first onset of other mental disorders** | | | | | | | | | | | | | | | | | |
| --- | --- | --- | --- | --- | --- | --- | --- | --- | --- | --- | --- | --- | --- | --- | --- | --- | --- |
|  |  |  | **PTSD** | | |  | **ADHD** | | |  | **AUD** | | |  | **DUD** | | |
| **Predictor** | **Timing** |  | **RR** | **Lower bound** | **Upper bound** |  | **RR** | **Lower bound** | **Upper bound** |  | **RR** | **Lower bound** | **Upper bound** |  | **RR** | **Lower bound** | **Upper bound** |
| MDE | Prior |  | 2.8 | 2.7 | 2.9 |  | 1.1 | 0.8 | 1.4 |  | 1.4 | 1.3 | 1.5 |  | 2.0 | 1.8 | 2.1 |
|  | Same |  | 4.2 | 3.9 | 4.4 |  | 3.7 | 3.0 | 4.5 |  | 1.8 | 1.6 | 2.0 |  | 3.2 | 2.8 | 3.6 |
| M/HM | Prior |  | 1.6 | 1.5 | 1.8 |  | 1.5 | 1.0 | 2.3 |  | 1.5 | 1.3 | 1.7 |  | 1.6 | 1.4 | 1.8 |
|  | Same |  | 1.9 | 1.7 | 2.2 |  | 2.0 | 1.2 | 3.2 |  | 2.4 | 2.0 | 2.8 |  | 2.2 | 1.8 | 2.7 |
| Panic disorder | Prior |  | 1.5 | 1.4 | 1.7 |  | 0.9 | 0.6 | 1.3 |  | 1.1 | 1.0 | 1.2 |  | 1.2 | 1.0 | 1.3 |
|  | Same |  | 1.8 | 1.7 | 2.0 |  | 1.9 | 1.4 | 2.8 |  | 1.4 | 1.1 | 1.7 |  | 2.0 | 1.7 | 2.5 |
| GAD | Prior |  | 1.6 | 1.5 | 1.7 |  | 2.6 | 2.1 | 3.3 |  | 0.9 | 0.8 | 1.0 |  | 1.1 | 1.0 | 1.2 |
|  | Same |  | 2.5 | 2.4 | 2.7 |  | 4.4 | 3.6 | 5.5 |  | 1.3 | 1.1 | 1.5 |  | 1.8 | 1.6 | 2.2 |
| PTSD | Prior |  | - | - | - |  | 1.2 | 0.9 | 1.7 |  | 1.5 | 1.4 | 1.6 |  | 1.5 | 1.4 | 1.7 |
|  | Same |  | - | - | - |  | 1.8 | 1.3 | 2.4 |  | 2.1 | 2.0 | 2.3 |  | 1.9 | 1.7 | 2.3 |
| ADHD | Prior |  | 1.3 | 1.3 | 1.4 |  | - | - | - |  | 1.4 | 1.3 | 1.5 |  | 1.6 | 1.4 | 1.7 |
|  | Same |  | 1.4 | 1.0 | 1.8 |  | - | - | - |  | 0.2 | 0.1 | 0.5 |  | 0.2 | 0.1 | 0.7 |
| Alcohol | Prior |  | 1.4 | 1.3 | 1.5 |  | 0.6 | 0.3 | 1.0 |  | - | - | - |  | 5.1 | 4.5 | 5.7 |
|  | Same |  | 1.9 | 1.8 | 2.1 |  | 0.3 | 0.1 | 0.9 |  | - | - | - |  | 8.3 | 7.4 | 9.2 |
| Drugs | Prior |  | 1.2 | 1.1 | 1.3 |  | 0.5 | 0.2 | 1.1 |  | 1.6 | 1.4 | 1.8 |  | - | - | - |
|  | Same |  | 1.5 | 1.4 | 1.8 |  | 0.4 | 0.1 | 1.2 |  | 6.4 | 5.8 | 7.0 |  | - | - | - |
|  |  |  |  |  |  |  |  |  |  |  |  |  |  |  |  |  |  |
| **Significance tests of predictors** | |  | **F-value** | **p-value** | **Numerator DF** |  | **F-value** | **p-value** | **Numerator DF** |  | **F-value** | **p-value** | **Numerator DF** |  | **F-value** | **p-value** | **Numerator DF** |
| All dx | Prior |  | 970.1 | <0.001 | 7 |  | 13.4 | <0.001 | 7 |  | 128.0 | <0.001 | 7 |  | 417.9 | <0.001 | 7 |
| All dx | Same |  | 813.8 | <0.001 | 7 |  | 129.6 | <0.001 | 7 |  | 345.2 | <0.001 | 7 |  | 439.5 | <0.001 | 7 |
|  |  |  |  |  |  |  |  |  |  |  |  |  |  |  |  |  |  |

ADHD, attention deficit/hyperactivity disorder; AUD, alcohol use disorder; DUD, drug use disorder; GAD, generalized anxiety disorder; MDE, major depressive episode; M/HM, mania or hypomania, PTSD, post-traumatic stress disorder; RR, risk ratio
