# Supplementary material for: Comorbidity and temporal associations between mental disorders among college students in the world mental health international college student initiative
Source: Psychiatry Res. Author manuscript; Available in PMC 2026 May 18. (PMC13181139; doi:10.1016/j.psychres.2025.116605)
Supplement: 4 [file NIHMS2168631-supplement-4.docx]

| **Supplementary Table 4. Temporally primary disorders (prior or same age of onset) and scales (based on either prior or same year onset mental disorders) and subsequent first onset of other mental disorders**  **Model 3** | | | | | | | | | | | | | | | | | |
| --- | --- | --- | --- | --- | --- | --- | --- | --- | --- | --- | --- | --- | --- | --- | --- | --- | --- |
|  |  |  | **MDE** | | |  | **Mania/Hypomania** | | |  | **Panic** | | |  | **GAD** | | |
| **Predictor** | **Timing** |  | **RR** | **Lower bound** | **Upper bound** |  | **RR** | **Lower bound** | **Upper bound** |  | **RR** | **Lower bound** | **Upper bound** |  | **RR** | **Lower bound** | **Upper bound** |
| MDE | Prior |  | - | - | - |  | 3.5 | 3.0 | 4.1 |  | 3.3 | 2.9 | 3.7 |  | 5.8 | 5.3 | 6.3 |
|  | Same |  | - | - | - |  | 3.9 | 3.2 | 4.7 |  | 4.9 | 4.3 | 5.6 |  | 28.7 | 26.7 | 30.8 |
| M/HM | Prior |  | 1.5 | 1.3 | 1.7 |  | - | - | - |  | 1.6 | 1.3 | 1.9 |  | 1.2 | 1.0 | 1.4 |
|  | Same |  | 2.3 | 1.9 | 2.6 |  | - | - | - |  | 2.2 | 1.8 | 2.8 |  | 1.8 | 1.5 | 2.1 |
| Panic disorder | Prior |  | 1.8 | 1.6 | 2.0 |  | 2.7 | 2.3 | 3.2 |  | - | - | - |  | 2.8 | 2.5 | 3.1 |
|  | Same |  | 3.9 | 3.5 | 4.3 |  | 2.7 | 2.1 | 3.6 |  | - | - | - |  | 7.8 | 6.9 | 8.7 |
| GAD | Prior |  | 4.4 | 4.1 | 4.8 |  | 2.2 | 1.9 | 2.7 |  | 4.4 | 3.8 | 5.1 |  | - | - | - |
|  | Same |  | 17.4 | 16.5 | 18.4 |  | 2.7 | 2.1 | 3.4 |  | 9.3 | 8.1 | 10.6 |  | - | - | - |
| PTSD | Prior |  | 2.2 | 2.1 | 2.4 |  | 2.8 | 2.4 | 3.3 |  | 2.5 | 2.2 | 2.8 |  | 2.2 | 2.0 | 2.4 |
|  | Same |  | 4.8 | 4.5 | 5.1 |  | 2.7 | 2.3 | 3.3 |  | 2.8 | 2.4 | 3.2 |  | 4.6 | 4.2 | 5.0 |
| ADHD | Prior |  | 2.0 | 1.8 | 2.1 |  | 1.6 | 1.5 | 1.8 |  | 1.4 | 1.3 | 1.6 |  | 1.6 | 1.5 | 1.7 |
|  | Same |  | 2.2 | 1.9 | 2.6 |  | 1.7 | 1.1 | 2.8 |  | 1.9 | 1.3 | 2.7 |  | 4.0 | 3.3 | 4.8 |
| Alcohol | Prior |  | 1.1 | 1.0 | 1.2 |  | 1.5 | 1.3 | 1.7 |  | 1.0 | 0.9 | 1.2 |  | 0.9 | 0.8 | 1.0 |
|  | Same |  | 1.7 | 1.5 | 1.9 |  | 2.6 | 2.2 | 3.1 |  | 1.4 | 1.1 | 1.8 |  | 1.3 | 1.1 | 1.5 |
| Drugs | Prior |  | 1.0 | 0.9 | 1.1 |  | 1.2 | 1.0 | 1.4 |  | 1.1 | 0.9 | 1.3 |  | 1.0 | 0.9 | 1.2 |
|  | Same |  | 2.3 | 2.0 | 2.6 |  | 2.2 | 1.7 | 2.7 |  | 2.0 | 1.6 | 2.4 |  | 1.7 | 1.5 | 2.0 |
| Internalizing scale | Prior |  | 0.7 | 0.7 | 0.7 |  | 0.7 | 0.6 | 0.7 |  | 0.7 | 0.6 | 0.7 |  | 0.7 | 0.7 | 0.8 |
|  | Same |  | 0.6 | 0.5 | 0.6 |  | 0.8 | 0.7 | 0.9 |  | 0.6 | 0.6 | 0.7 |  | 0.6 | 0.6 | 0.6 |
| Externalizing scale | Prior |  | 0.7 | 0.6 | 0.8 |  | - | - | - |  | 0.8 | 0.7 | 0.9 |  | 0.8 | 0.6 | 0.9 |
|  | Same |  | 1.2 | 0.8 | 1.7 |  | - | - | - |  | 1.2 | 0.5 | 2.9 |  | 0.8 | 0.5 | 1.3 |
| Substance use scale | Prior |  | 1.0 | 0.9 | 1.1 |  | 1.0 | 0.8 | 1.1 |  | 1.0 | 0.8 | 1.1 |  | 1.0 | 0.9 | 1.1 |
|  | Same |  | 0.9 | 0.7 | 1.1 |  | 0.9 | 0.7 | 1.1 |  | 0.8 | 0.6 | 1.1 |  | 0.7 | 0.6 | 1.0 |
|  |  |  |  |  |  |  |  |  |  |  |  |  |  |  |  |  |  |
| **Significance tests of predictors** | |  | **F-value** | **p-value** | **Numerator DF** |  | **F-value** | **p-value** | **Numerator DF** |  | **F-value** | **p-value** | **Numerator DF** |  | **F-value** | **p-value** | **Numerator DF** |
| All dx | Prior |  | 378.3 | <0.001 | 7 |  | 78.1 | <0.001 | 7 |  | 117.5 | <0.001 | 7 |  | 231.4 | <0.001 | 7 |
| All dx | Same |  | 1843.1 | <0.001 | 7 |  | 86.4 | <0.001 | 7 |  | 237.9 | <0.001 | 7 |  | 1471.5 | <0.001 | 7 |
| All scales | Prior |  | 58.9 | <0.001 | 3 |  | 30.6 | <0.001 | 2 |  | 36.4 | <0.001 | 3 |  | 34.0 | <0.001 | 3 |
| All scales | Same |  | 149.0 | <0.001 | 3 |  | 7.1 | <0.001 | 2 |  | 26.8 | <0.001 | 3 |  | 84.9 | <0.001 | 3 |
|  |  |  |  |  |  |  |  |  |  |  |  |  |  |  |  |  |  |

Abbreviations: ADHD, attention deficit/hyperactivity disorder; AUD, alcohol use disorder; DUD, drug use disorder; GAD, generalized anxiety disorder; MDE, major depressive episode; M/HM, mania or hypomania; PD, panic disorder; PTSD, post-traumatic stress disorder; RR, risk ratio

| **Supplementary Table 4 (continued). Temporally primary disorders (prior or same age of onset) and scales (based on either prior or same year onset mental disorders) and subsequent first onset of other mental disorders** | | | | | | | | | | | | | | | | | |
| --- | --- | --- | --- | --- | --- | --- | --- | --- | --- | --- | --- | --- | --- | --- | --- | --- | --- |
|  |  |  | **PTSD** | | |  | **ADHD** | | |  | **AUD** | | |  | **DUD** | | |
| **Predictor** | **Timing** |  | **RR** | **Lower bound** | **Upper bound** |  | **RR** | **Lower bound** | **Upper bound** |  | **RR** | **Lower bound** | **Upper bound** |  | **RR** | **Lower bound** | **Upper bound** |
| MDE | Prior |  | 3.2 | 3.0 | 3.3 |  | 1.7 | 1.3 | 2.2 |  | 1.8 | 1.6 | 1.9 |  | 2.6 | 2.3 | 2.9 |
|  | Same |  | 4.4 | 4.1 | 4.7 |  | 5.2 | 4.3 | 6.4 |  | 2.1 | 1.8 | 2.3 |  | 3.7 | 3.2 | 4.2 |
| M/HM | Prior |  | 1.8 | 1.6 | 1.9 |  | 1.6 | 1.0 | 2.4 |  | 1.6 | 1.4 | 1.8 |  | 1.6 | 1.4 | 1.9 |
|  | Same |  | 1.9 | 1.7 | 2.2 |  | 2.0 | 1.3 | 3.2 |  | 2.4 | 2.0 | 2.8 |  | 2.2 | 1.8 | 2.7 |
| Panic disorder | Prior |  | 2.3 | 2.1 | 2.4 |  | 1.5 | 0.9 | 2.3 |  | 1.5 | 1.3 | 1.7 |  | 1.7 | 1.4 | 2.0 |
|  | Same |  | 2.3 | 2.0 | 2.5 |  | 3.1 | 2.2 | 4.5 |  | 1.6 | 1.3 | 2.0 |  | 2.5 | 2.0 | 3.1 |
| GAD | Prior |  | 2.7 | 2.5 | 2.9 |  | 3.8 | 3.0 | 4.8 |  | 1.3 | 1.1 | 1.4 |  | 1.7 | 1.4 | 2.0 |
|  | Same |  | 3.5 | 3.2 | 3.8 |  | 7.0 | 5.7 | 8.7 |  | 1.7 | 1.4 | 2.0 |  | 2.6 | 2.1 | 3.2 |
| PTSD | Prior |  | - | - | - |  | 1.5 | 1.1 | 2.1 |  | 1.7 | 1.6 | 1.8 |  | 1.9 | 1.7 | 2.2 |
|  | Same |  | - | - | - |  | 2.2 | 1.6 | 3.0 |  | 2.2 | 2.0 | 2.4 |  | 2.1 | 1.8 | 2.5 |
| ADHD | Prior |  | 1.4 | 1.3 | 1.4 |  | - | - | - |  | 1.4 | 1.3 | 1.5 |  | 1.6 | 1.4 | 1.7 |
|  | Same |  | 1.4 | 1.0 | 1.8 |  | - | - | - |  | 0.2 | 0.1 | 0.5 |  | 0.2 | 0.1 | 0.7 |
| AUD | Prior |  | 1.4 | 1.4 | 1.5 |  | 0.6 | 0.3 | 1.0 |  | - | - | - |  | 5.0 | 4.5 | 5.7 |
|  | Same |  | 2.0 | 1.8 | 2.1 |  | 0.3 | 0.1 | 0.9 |  | - | - | - |  | 8.1 | 7.3 | 9.1 |
| DUD | Prior |  | 1.3 | 1.2 | 1.4 |  | 0.5 | 0.2 | 1.6 |  | 1.6 | 1.4 | 1.8 |  | - | - | - |
|  | Same |  | 1.6 | 1.4 | 1.9 |  | 0.4 | 0.1 | 1.3 |  | 6.3 | 5.7 | 6.9 |  | - | - | - |
| Internalizing scale | Prior |  | 0.7 | 0.7 | 0.7 |  | 0.6 | 0.5 | 0.7 |  | 0.8 | 0.7 | 0.8 |  | 0.7 | 0.7 | 0.8 |
|  | Same |  | 0.7 | 0.7 | 0.8 |  | 0.6 | 0.5 | 0.7 |  | 0.8 | 0.7 | 0.9 |  | 0.7 | 0.6 | 0.8 |
| Externalizing scale | Prior |  | 0.8 | 0.7 | 0.9 |  | - | - | - |  | 0.9 | 0.8 | 1.0 |  | 1.0 | 0.8 | 1.1 |
|  | Same |  | 0.9 | 0.4 | 1.9 |  | - | - | - |  | 0.0 | 0.0 | 0.6 |  | 2.8 | 0.8 | 9.9 |
| Substance use scale | Prior |  | 0.9 | 0.8 | 1.0 |  | 0.8 | 0.3 | 2.6 |  | - | - | - |  | - | - | - |
|  | Same |  | 0.8 | 0.6 | 0.9 |  | 0.0 | 0.0 | 1.2 |  | - | - | - |  | - | - | - |
|  |  |  |  |  |  |  |  |  |  |  |  |  |  |  |  |  |  |
| **Significance tests of predictors** | |  | **F-value** | **p-value** | **Numerator DF** |  | **F-value** | **p-value** | **Numerator DF** |  | **F-value** | **p-value** | **Numerator DF** |  | **F-value** | **p-value** | **Numerator DF** |
| All dx | Prior |  | 526.5 | <0.001 | 7 |  | 16.2 | <0.001 | 7 |  | 94.3 | <0.001 | 7 |  | 255.6 | <0.001 | 7 |
| All dx | Same |  | 469.1 | <0.001 | 7 |  | 67.1 | <0.001 | 7 |  | 296.4 | <0.001 | 7 |  | 332.4 | <0.001 | 7 |
| All scales | Prior |  | 91.1 | <0.001 | 3 |  | 14.7 | <0.001 | 2 |  | 39.4 | <0.001 | 2 |  | 21.7 | <0.001 | 2 |
| All scales | Same |  | 41.9 | <0.001 | 3 |  | 5.3 | 0.01 | 2 |  | 5.9 | 0.01 | 2 |  | 16.1 | <0.001 | 2 |
|  |  |  |  |  |  |  |  |  |  |  |  |  |  |  |  |  |  |

ADHD, attention deficit/hyperactivity disorder; AUD, alcohol use disorder; DUD, drug use disorder; GAD, generalized anxiety disorder; MDE, major depressive episode; M/HM, mania or hypomania; PD, panic disorder; PTSD, post-traumatic stress disorder; RR, risk ratio
