# Supplementary material for: Comorbidity and temporal associations between mental disorders among college students in the world mental health international college student initiative
Source: Psychiatry Res. Author manuscript; Available in PMC 2026 May 18. (PMC13181139; doi:10.1016/j.psychres.2025.116605)
Supplement: 5 [file NIHMS2168631-supplement-5.docx]

| **Supplementary Table 5. Temporally primary disorders (prior or same age of onset combined) and persistence of other mental disorders**  **Model 2** | | | | | | | | | | | | | | | | | | | | | | | | | |
| --- | --- | --- | --- | --- | --- | --- | --- | --- | --- | --- | --- | --- | --- | --- | --- | --- | --- | --- | --- | --- | --- | --- | --- | --- | --- |
|  | |  | **Outcome disorder** | | | | | | | | | | | | | | | | | | | | | | |
|  | |  | **MDE** | | |  | **Mania/hypomania** | | |  | **Panic** | | |  | **GAD** | | |  | **PTSD** | | |  | **Drugs** | | |
| **Predictor** | **Timing** |  | **RR** | **Low RR** | **Upper RR** |  | **RR** | **Low RR** | **Upper RR** |  | **RR** | **Low RR** | **Upper RR** |  | **RR** | **Low RR** | **Upper RR** |  | **RR** | **Low RR** | **Upper RR** |  | **RR** | **Low RR** | **Upper RR** |
| MDE | Prior or same AOO |  | - | - | - |  | 1.05 | 0.98 | 1.13 |  | 1.09 | 1.04 | 1.13 |  | 1.03 | 1.01 | 1.06 |  | 1.06 | 1.03 | 1.09 |  | 0.97 | 0.91 | 1.03 |
| M/HM | Prior or same AOO |  | 1.02 | 0.99 | 1.06 |  | - | - | - |  | 1.07 | 0.99 | 1.16 |  | 1.05 | 1.01 | 1.10 |  | 1.12 | 1.06 | 1.18 |  | 1.08 | 0.98 | 1.19 |
| Panic disorder | Prior or same AOO |  | 1.05 | 1.04 | 1.07 |  | 1.11 | 1.03 | 1.20 |  | - | - | - |  | 1.02 | 0.99 | 1.06 |  | 1.06 | 1.02 | 1.11 |  | 0.96 | 0.87 | 1.06 |
| GAD | Prior or same AOO |  | 1.02 | 1.01 | 1.04 |  | 1.04 | 0.97 | 1.12 |  | 1.05 | 1.01 | 1.10 |  | - | - | - |  | 1.09 | 1.06 | 1.13 |  | 1.04 | 0.97 | 1.12 |
| PTSD | Prior or same AOO |  | 1.02 | 1.01 | 1.04 |  | 1.09 | 1.00 | 1.19 |  | 1.05 | 1.00 | 1.09 |  | 1.02 | 0.99 | 1.04 |  | - | - | - |  | 1.02 | 0.96 | 1.09 |
| ADHD | Prior or same AOO |  | 1.05 | 1.03 | 1.07 |  | 1.14 | 1.06 | 1.23 |  | 1.05 | 1.01 | 1.09 |  | 1.05 | 1.03 | 1.08 |  | 1.08 | 1.05 | 1.11 |  | 1.07 | 1.00 | 1.14 |
| Alcohol | Prior or same AOO |  | 1.01 | 0.98 | 1.04 |  | 1.06 | 0.95 | 1.18 |  | 1.02 | 0.95 | 1.10 |  | 1.03 | 0.99 | 1.08 |  | 1.04 | 0.99 | 1.09 |  | 1.17 | 1.11 | 1.24 |
| Drugs | Prior or same AOO |  | 1.00 | 0.96 | 1.03 |  | 0.98 | 0.86 | 1.11 |  | 1.04 | 0.95 | 1.14 |  | 1.00 | 0.95 | 1.05 |  | 1.00 | 0.95 | 1.06 |  | - | - | - |
| Tests for significance of all prior or same-year dx predictors | F/p-val/DF |  | 17.03 | <0.001 | 7 |  | 8.05 | <0.001 | 7 |  | 9.29 | <0.001 | 7 |  | 6.86 | <0.001 | 7 |  | 25.23 | <0.001 | 7 |  | 6.87 | <0.001 | 7 |
|  |  |  |  |  |  |  |  |  |  |  |  |  |  |  |  |  |  |  |  |  |  |  |  |  |  |

ADHD, attention deficit/hyperactivity disorder; AUD, alcohol use disorder; DUD, drug use disorder; GAD, generalized anxiety disorder; MDE, major depressive episode; M/HM, mania or hypomania; PD, panic disorder; PTSD, post-traumatic stress disorder; RR, risk ratio
