# Supplementary material for: Comorbidity and temporal associations between mental disorders among college students in the world mental health international college student initiative
Source: Psychiatry Res. Author manuscript; Available in PMC 2026 May 18. (PMC13181139; doi:10.1016/j.psychres.2025.116605)
Supplement: 6 [file NIHMS2168631-supplement-6.docx]

| **Supplementary Table 6. Temporally primary disorders (prior or same age of onset combined) and persistence of other mental disorders**  **Model 3** | | | | | | | | | | | | | | | | | | | | | | | | | |
| --- | --- | --- | --- | --- | --- | --- | --- | --- | --- | --- | --- | --- | --- | --- | --- | --- | --- | --- | --- | --- | --- | --- | --- | --- | --- |
|  | |  | **outcome disorder** | | | | | | | | | | | | | | | | | | | | | | |
|  | |  | **MDE** | | |  | **Mania/hypomania** | | |  | **Panic** | | |  | **GAD** | | |  | **PTSD** | | |  | **Drugs** | | |
| **Predictor** | **Timing** |  | **RR** | **Low RR** | **Upper RR** |  | **RR** | **Low RR** | **Upper RR** |  | **RR** | **Low RR** | **Upper RR** |  | **RR** | **Low RR** | **Upper RR** |  | **RR** | **Low RR** | **Upper RR** |  | **RR** | **Low RR** | **Upper RR** |
| MDE | Prior or same |  | - | - | - |  | 1.10 | 1.00 | 1.22 |  | 1.12 | 1.05 | 1.19 |  | 1.04 | 1.01 | 1.07 |  | 1.08 | 1.04 | 1.12 |  | 0.97 | 0.89 | 1.05 |
| M/HM | Prior or same |  | 1.03 | 0.99 | 1.07 |  | - | - | - |  | 1.08 | 0.98 | 1.18 |  | 1.06 | 1.01 | 1.12 |  | 1.13 | 1.06 | 1.20 |  | 1.06 | 0.94 | 1.19 |
| Panic disorder | Prior or same |  | 1.06 | 1.03 | 1.09 |  | 1.16 | 1.04 | 1.31 |  | - | - | - |  | 1.03 | 0.99 | 1.07 |  | 1.09 | 1.03 | 1.15 |  | 0.96 | 0.85 | 1.08 |
| GAD | Prior or same |  | 1.03 | 1.01 | 1.05 |  | 1.11 | 0.98 | 1.24 |  | 1.09 | 1.03 | 1.16 |  | - | - | - |  | 1.13 | 1.08 | 1.19 |  | 1.04 | 0.92 | 1.16 |
| PTSD | Prior or same |  | 1.03 | 1.01 | 1.05 |  | 1.13 | 1.02 | 1.25 |  | 1.07 | 1.01 | 1.13 |  | 1.02 | 0.99 | 1.06 |  | - | - | - |  | 1.02 | 0.94 | 1.10 |
| ADHD | Prior or same |  | 1.05 | 1.04 | 1.07 |  | 1.14 | 1.06 | 1.23 |  | 1.05 | 1.00 | 1.10 |  | 1.06 | 1.03 | 1.08 |  | 1.08 | 1.05 | 1.12 |  | 1.06 | 0.99 | 1.13 |
| Alcohol | Prior or same |  | 1.00 | 0.97 | 1.04 |  | 1.05 | 0.94 | 1.17 |  | 1.03 | 0.94 | 1.12 |  | 1.04 | 0.98 | 1.09 |  | 1.04 | 0.98 | 1.10 |  | 1.17 | 1.11 | 1.24 |
| Drugs | Prior or same |  | 0.98 | 0.93 | 1.02 |  | 0.96 | 0.80 | 1.14 |  | 1.05 | 0.94 | 1.18 |  | 1.00 | 0.94 | 1.07 |  | 1.01 | 0.94 | 1.08 |  | - | - | - |
| Internalizing scale | Prior or same |  | 0.99 | 0.98 | 1.01 |  | 0.95 | 0.89 | 1.03 |  | 0.97 | 0.93 | 1.01 |  | 0.99 | 0.97 | 1.02 |  | 0.97 | 0.94 | 1.00 |  | 1.00 | 0.94 | 1.07 |
| Externalizing scale | Prior or same |  | 0.98 | 0.94 | 1.02 |  |  |  |  |  | 0.99 | 0.92 | 1.07 |  | 0.98 | 0.93 | 1.03 |  | 0.98 | 0.93 | 1.04 |  | 1.04 | 0.94 | 1.15 |
| Substance use scale | Prior or same |  | 1.03 | 0.99 | 1.06 |  | 1.03 | 0.92 | 1.16 |  | 0.99 | 0.90 | 1.08 |  | 0.99 | 0.94 | 1.05 |  | 1.00 | 0.93 | 1.06 |  |  |  |  |
| Tests for significance of all dx predictors | F/p-val/DF |  | 8.89 | <0.001 | 7 |  | 3.52 | <0.001 | 7 |  | 3.67 | <0.001 | 7 |  | 4.77 | <0.001 | 7 |  | 12.18 | <0.001 | 7 |  | 5.69 | <0.001 | 7 |
| Tests for significance of all scales | F/p-val/DF |  | 1.19 | 0.31101 | 3 |  | .98 | 0.37822 | 2 |  | 0.71 | 0.54577 | 3 |  | 0.35 | 0.78831 | 3 |  | 1.40 | 0.24233 | 3 |  | 0.27 | 0.76665 | 2 |
|  |  |  |  |  |  |  |  |  |  |  |  |  |  |  |  |  |  |  |  |  |  |  |  |  |  |

ADHD, attention deficit/hyperactivity disorder; AUD, alcohol use disorder; DUD, drug use disorder; GAD, generalized anxiety disorder; MDE, major depressive episode; M/HM, mania or hypomania; D, panic disorder; PTSD, post-traumatic stress disorder; RR, risk ratio
